# Oxidative stress-related biomarkers as promising indicators of inflammatory bowel disease activity: A systematic review and meta-analysis

**DOI:** 10.1016/j.redox.2024.103380

**Published:** 2024-10-01

**Authors:** Armando Tratenšek, Igor Locatelli, Iztok Grabnar, David Drobne, Tomaž Vovk

**Affiliations:** a University of Ljubljana, Faculty of Pharmacy, Aškerčeva cesta 7, 1000 Ljubljana, Slovenia; b University Medical Centre Ljubljana, Department of Gastroenterology, Japljeva ulica 2, 1000 Ljubljana, Slovenia; c University of Ljubljana, Faculty of Medicine, Vrazov trg 2, 1000 Ljubljana, Slovenia

**Keywords:** Inflammatory bowel disease, Ulcerative colitis, Crohn's disease, Oxidative stress, Biomarker, Meta-analysis

## Abstract

Oxidative stress is believed to play an important role in the pathogenesis of inflammatory bowel disease (IBD), specifically Crohn's disease (CD) and ulcerative colitis (UC). This meta-analysis aimed to identify and quantify the oxidative stress-related biomarkers in IBD and their associations with disease activity. We systematically searched Ovid MEDLINE, Ovid Embase, and Web of Science databases, identifying 54 studies for inclusion. Comparisons included: (i) active IBD versus healthy controls; (ii) inactive IBD versus healthy controls; (iii) active CD versus inactive CD; and (iv) active UC versus inactive UC. Our analysis revealed a significant accumulation of biomarkers of oxidative damage to biomacromolecules, coupled with reductions in various antioxidants, in both patients with active and inactive IBD compared to healthy controls. Additionally, we identified biomarkers that differentiate between active and inactive CD, including malondialdehyde, Paraoxonase 1, catalase, albumin, transferrin, and total antioxidant capacity. Similarly, levels of Paraoxonase 1, erythrocyte glutathione peroxidase, catalase, albumin, transferrin, and free thiols differed between active and inactive UC. Vitamins and carotenoids also emerged as potential disease activity biomarkers for CD and UC, but their intake should be monitored to obtain meaningful results. These findings emphasize the involvement of oxidative stress in the pathogenesis of IBD and highlight the potential of oxidative stress-related biomarkers as a minimally invasive and additional tool for monitoring the activity of IBD.

## Highlights

• Reduced antioxidants and accumulated oxidative damage indicate oxidative stress in IBD.
• Oxidative stress is more pronounced in active than in inactive disease.
• Despite IBD inactivity, oxidative stress-related biomarkers remain dysregulated.
• MDA, PON-1, CAT, albumin, transferrin, and TAC indicate Crohn's disease flares.
• PON-1, GPx, CAT, albumin, transferrin, and R–SH indicate ulcerative colitis flares.

## Graphical abstract

**Figure:**
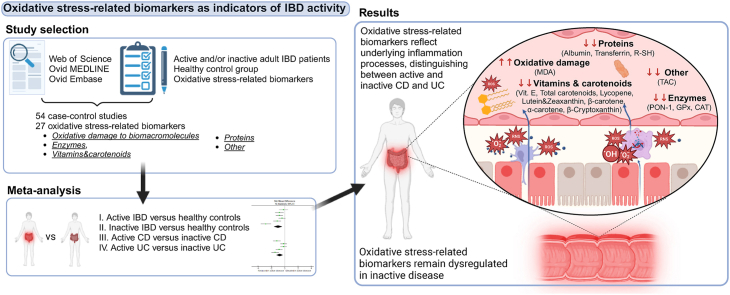

## 1 Introduction

Inflammatory Bowel Disease (IBD), encompassing Crohn's disease (CD) and ulcerative colitis (UC), is characterized by chronic and relapsing inflammation of the gastrointestinal tract. The complex etiology of IBD involves intricate interactions between genetic, environmental, and immunological factors [1,2]. In recent years, oxidative stress, defined as an imbalance between oxidants and antioxidants in favour of the former [3], has emerged as a pivotal player in the pathogenesis of IBD and is associated with disease activity, progression, and clinical outcomes [4,5]. The chronically inflamed intestinal mucosa is prone to the excessive production of reactive oxygen and nitrogen species by activated immune cells, such as macrophages and neutrophils. These reactive species induce lipid peroxidation, protein modifications, and DNA damage, which collectively contribute to tissue injury, impaired mucosal healing, and perpetuated inflammation. In CD, the inflammation extends transmurally across all layers of the intestinal wall, while in UC, it is typically confined to the mucosal layer [[4], [5], [6], [7]].

IBD is characterized by alternating periods of remission and exacerbations, with patients experiencing fluctuations in disease activity [1,2]. However, clinical symptoms are often not consistent with more objective measures of inflammation, such as endoscopic activity and levels of C-reactive protein [8]. Currently, achieving endoscopic healing stands as the primary long-term treatment target for managing IBD. It is believed that reaching this goal helps prevent long-term disease-related complications such as bowel damage, hospitalisations, surgeries, and intestinal failure [9]. However, persistent histological and immune-related gene expression abnormalities were found in patients with the endoscopically inactive disease [10]. This led researchers to explore whether the concept of disease clearance (symptomatically, endoscopically, and histologically inactive disease) and molecular healing could be the ultimate treatment goal [11].

In clinical practice, there is no single “gold standard” test or examination and physicians apply a combination of clinical symptoms, laboratory parameters, endoscopy, imaging, and histological examination to accurately assess the disease activity. This is crucial for tailoring therapeutic interventions and evaluating treatment efficacy [12,13].

To minimize the need for invasive endoscopic procedures and obtain more objective measurements of disease activity, new laboratory markers are being studied as an additive tool in the management of IBD. The integration of various biomarkers could facilitate the early identification of patients susceptible to disease relapse, allowing for enhanced surveillance and proactive optimization of treatment strategies [14,15].

Oxidative stress-related biomarkers offer a novel and minimally invasive approach for assessing disease activity [16]. They can be detected not only in the inflamed intestinal mucosa [[17], [18], [19]] but also in the peripheral blood [[20], [21], [22], [23], [24], [25], [26]]. These biomarkers encompass products of lipid peroxidation, protein oxidation, oxidative DNA damage, and altered levels of antioxidants [21]. Several studies have found differences in the levels of biomarkers related to oxidative stress in patients with active disease, patients in remission, and healthy controls, highlighting their potential as surrogate markers for active mucosal inflammation [23,24,[26], [27], [28]]. However, the specific biomarkers that show the strongest association with disease activity and the ability to detect IBD exacerbations are still unknown. Also, little is known about the extent of oxidative stress in inactive disease compared to healthy individuals.

The aim of this comprehensive systematic review and meta-analysis was to clarify the associations between oxidative stress-related biomarkers and IBD activity by comparing their levels in patients with active disease, inactive disease, and healthy individuals. The study also aimed to investigate potential differences between UC and CD.

## 2 Materials and methods

### 2.1 Protocol registration

The present systematic review and meta-analysis complied with the Preferred Reporting Items for Systematic Reviews and Meta-Analyses (PRISMA) guidelines [29]. The study protocol was registered in the International Prospective Register of Systematic Reviews database (PROSPERO; CRD42021285456).

### 2.2 Search strategy

Bibliographic databases, including Ovid MEDLINE, Ovid Embase, and Web of Science, were searched for relevant adult human studies written in the English language from January 1, 2000, to May 10, 2024. The search terms and operators used were: (“oxidative stress” OR “oxidative damage” OR “antioxida∗") AND (“Crohn's disease” OR “ulcerative colitis” OR “inflammatory bowel disease” OR “IBD”) AND (“marker∗” OR “index” OR “parameter∗” OR “biomarker∗”). The full literature search strategy is provided in the Supplementary Table 1. References of previously-published systematic reviews and meta-analyses focusing on oxidative stress-related biomarkers in inflammatory bowel disease were manually searched for any additional records.

### 2.3 Inclusion criteria

This review covered studies that investigated differences in the levels of oxidative stress-related biomarkers between patients with IBD and healthy controls, as well as their association with disease activity. The inclusion criteria were defined in accordance with the ‘population, exposure, comparison, outcomes and study design’ (PECOS) model as follows: (i) Population; adult patients with inflammatory bowel disease (Crohn's disease and ulcerative colitis) and healthy controls. (ii) Exposure; any available biomarker of oxidative stress, including biomarkers of oxidative damage to biomacromolecules (proteins, DNA, lipids) and levels of various antioxidants (enzymatic, vitamins and carotenoids, proteins, other). (iii) Comparison; levels of oxidative stress-related biomarkers in healthy, preferably age- and sex-matched controls. (iv) Outcome; association of the selected oxidative stress-related biomarkers with inflammatory bowel disease and its activity. The outcomes had to be reported as the mean and standard deviation for both the investigated (active or inactive) group and healthy controls. Alternatively, sufficient data had to be available to estimate the mean and standard deviation. (v) Study design; population-based observational case-control studies (cross-sectional and longitudinal).

### 2.4 Exclusion criteria

The following studies were excluded on the basis of predefined exclusion criteria: case reports, review articles, meta-analyses, conference abstracts; studies focusing on populations not relevant to the review (paediatric patients, patients with comorbidities); genetic and animal studies on oxidative stress; studies published before January 1, 2000; studies not written in English language; studies with inaccessible full-text; studies not related to markers of oxidative stress; studies without a healthy control group; studies where disease activity was not specified; and duplicated publications. Finally, biomarkers studied in fewer than three independent cohorts of both patients with active and inactive IBD were not analyzed. In some studies, results were reported separately for patients with CD and UC. This was considered as two independent cohorts within one study.

### 2.5 Study selection and data extraction

Potentially relevant studies were independently identified through title and abstract screening of articles retrieved in bibliographic searches by one of the authors (A. T.) and a collaborator specified in the PROSPERO database (N. A.). Subsequently, the results were cross-checked and re-evaluated by a second author (T. V.). The full texts of these studies were then retrieved and independently assessed by the two reviewers, excluding articles not meeting the eligibility criteria. In case of any disagreements, a third reviewer (I. L.) was available for discussion. Finally, the same authors extracted the following information from the included studies: first author; year of publication; country; number of cases (total, active disease, and remission) and controls; IBD subtype (Crohn's disease, ulcerative colitis, or both); disease activity index used to differentiate between the active and inactive disease states; sample type; and the mean (standard deviation or standard error), median (interquartile range), or median (min–max) levels of evaluated biomarkers in both cases (active/inactive) and controls. When it was not feasible to separately extract data for patients with active and inactive disease, the reviewers reached a consensus and classified the study cohort as “active” when the proportion of patients with active disease was at least 75 %, and vice versa for the “inactive” classification. For studies where data were only graphically represented, Digit software version 1.0.4 was utilized to extract the information. In cases where insufficient data were reported, the corresponding author was contacted by e-mail to obtain further details.

### 2.6 Data synthesis and statistical analysis

If necessary, data on biomarkers were first converted to mean and standard deviation using methods described by Hozo et al. [30] or the Cochrane Handbook [31]. When the outcomes were reported separately for mildly, moderately, and severely active disease, the subgroups were combined into a single active group, following the guidelines provided by the Cochrane Handbook [31].

To evaluate the differences in the levels of oxidative stress-related markers between patients with IBD (active or inactive state) and healthy controls, standardized mean difference (SMD) with a 95 % confidence interval (CI) was employed to express the measure of effect size. SMD was computed by subtracting the mean biomarker levels of the control group from those of the investigational group and then dividing the result by a pooled standard deviation across the two groups, incorporating an adjustment for small sample bias [32]. This approach standardizes the results of studies to a uniform scale, enabling direct comparisons within and between diverse groups of markers measured with different methods and expressed in various units [33]. According to Cohen, SMD values of 0.2, 0.5, and ≥0.8 represent small, moderate, and large effect sizes, respectively [34]. Between-study heterogeneity was calculated using Cochrane's Q and I-squared (I^2^) statistics. Due to the diversity of analytical methods, treatments, and patient characteristics, a conservative random-effects (DerSimonian and Laird method [35]) model was used to pool the SMDs. The significance of the overall effect size was assessed using Z-tests, and a p-value of <0.05 was considered statistically significant. The meta-analysis was stratified based on the disease activity, with each biomarker assessed as a new comparison. To reduce heterogeneity, only the results of studies conducted on the same type of biological sample were combined. SMDs and related statistics (active/inactive IBD versus healthy controls, active CD/UC versus inactive CD/UC) were computed using Review Manager (RevMan) version 5.4.1 (The Cochrane Collaboration Software, 2020).

When consistent analytical methods were used to measure biomarkers exhibiting the largest SMDs across at least five cohorts of patients with active IBD, further analyses were conducted to obtain clinically interpretable results. For this purpose, the mean difference was selected as the outcome measure when biomarker levels in the healthy control groups across studies varied by no more than 30 %; otherwise, the ratio of means was utilized. The pooled mean difference and ratio of means, along with their respective 95 % prediction intervals, were computed using the “meta” package of R software, version 4.1.0 within RStudio (RStudio, Boston, MA, USA). The methodologies employed for estimating between-study variance were consistent with those utilized in Review Manager.

### 2.7 Subgroup analyses

Subgroup analyses were conducted to directly compare the two primary subtypes of IBD, namely CD and UC, in their active and inactive states. These subgroup-analyses were limited to three-arm studies identified during the initial meta-analysis, featuring clearly defined IBD subtypes and accessible data for active and inactive disease states, aiming to minimize the analytical variability. The investigational group consisted of patients with active disease, while those with inactive disease represented the control group.

## 3 Results

### 3.1 Study selection

**Fig. 1:**
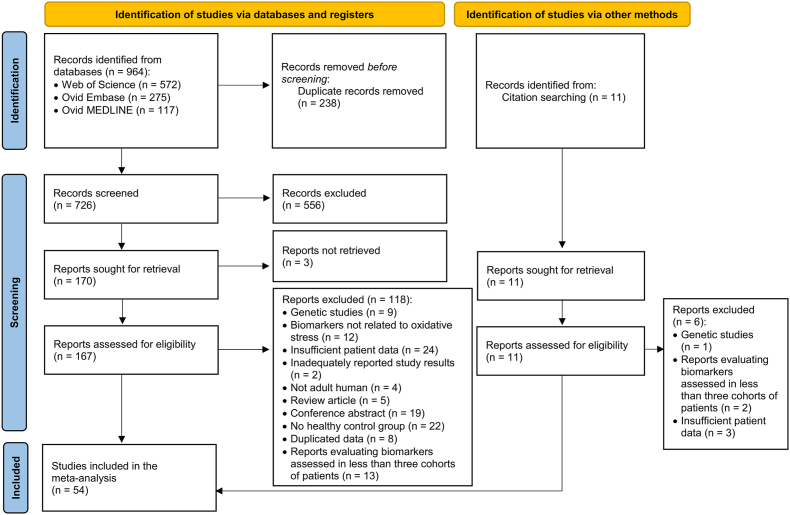
Flowchart of study identification and inclusion.

Through the initial database search, a total of 964 studies were identified. Following the removal of duplicated records, 726 studies were screened by title and abstract, resulting in the exclusion of an additional 556 studies. The remaining 170 studies were then sought for retrieval and a full-text evaluation was conducted for 167 of these studies, in addition to the 11 studies identified through the citation search. Finally, after excluding 109 studies, 69 remained eligible for inclusion in the systematic review. To meet the minimum requirement of having at least three cohorts of patients with active disease and at least three with inactive disease per biomarker, only 54 studies [[22], [23], [24],[26], [27], [28],[36], [37], [38], [39], [40], [41], [42], [43], [44], [45], [46], [47], [48], [49], [50], [51], [52], [53], [54], [55], [56], [57], [58], [59], [60], [61], [62], [63], [64], [65], [66], [67], [68], [69], [70], [71], [72], [73], [74], [75], [76], [77], [78], [79], [80], [81], [82], [83]] were subsequently included in the final meta-analysis (see Fig. 1).

### 3.2 Characteristics of included studies

Out of the 54 studies [[22], [23], [24],[26], [27], [28],[36], [37], [38], [39], [40], [41], [42], [43], [44], [45], [46], [47], [48], [49], [50], [51], [52], [53], [54], [55], [56], [57], [58], [59], [60], [61], [62], [63], [64], [65], [66], [67], [68], [69], [70], [71], [72], [73], [74], [75], [76], [77], [78], [79], [80], [81], [82], [83]] included in the meta-analysis, 23 studies [22,27,28,39,42,44,46,47,49,52,57,58,64,65,[67], [68], [69],[71], [72], [73], [74],^78^,^82^] focused exclusively on patients with CD, 9 studies [37,40,41,51,61,66,70,75,80] exclusively on patients with UC, and 18 studies [23,24,26,38,43,48,[53], [54], [55], [56],^59^,^62^,^63^,^76^,^77^,^79^,^81^,^83^] examined both CD and UC cohorts. Additionally, 4 studies [36,45,50,60] studies did not specify the IBD subtype and were categorized as IBD studies. The active and inactive disease states were compared to healthy controls in 48 and 40 studies, respectively. The sample size varied across the studies, ranging from 6 to 448 for subjects with active disease, 5 to 160 for those in remission, and 8 to 386 for the healthy control groups. The following oxidative stress-related biomarkers were evaluated the most: albumin (20 studies), malondialdehyde (MDA; 14 studies), glutathione peroxidase (GPx; 12 studies), plasma free thiols including cysteine and glutathione (R–SH; 10 studies), superoxide dismutase (SOD; 9 studies), total antioxidant capacity (TAC; 9 studies), selenium (8 studies), vitamin E (7 studies), β-carotene (7 studies), total bilirubin (TBIL; 6 studies), zinc (5 studies), vitamin C (5 studies), serum uric acid (SUA; 5 studies), catalase (4 studies), vitamin A (4 studies), advanced oxidation protein products (AOPP; 4 studies), Paraoxonase 1 (4 studies), transferrin (4 studies), lutein and zeaxanthin (3 studies), β-Cryptoxanthin (3 studies), lycopene (3 studies), α-carotene (3 studies), 8-iso-prostaglandin F2α (8-iso-PGF2α; 2 studies), total carotenoids (2 studies), and Selenoprotein P (2 studies). Further details are provided in Supplementary Table 2.

### 3.3 Meta-analysis

Individual comparisons were performed for each biomarker, evaluating the following populations: (i) Active IBD versus healthy controls; (ii) Inactive IBD versus healthy controls; (iii) Active CD versus inactive CD; and (iv) Active UC versus inactive UC.

#### 3.3.1 Active IBD versus healthy controls

In total, 48 studies compared the levels of 27 oxidative stress-related biomarkers between individuals with active IBD and healthy controls. Most studies assessed multiple biomarkers, and biological samples primarily consisted of plasma or serum, with the exception of GPx, catalase and SOD, which were also assessed in red and white blood cells. The number of subjects included in the analysis varied, ranging from 45 to 1297 for patients with active IBD, and from 45 to 1222 for healthy controls. Further details are provided in Table 1 and in Supplementary Fig. 1.

**Table 1:** Results of the meta-analysis of oxidative stress-related biomarkers in patients with active and inactive IBD versus healthy controls.

Group	Biomarker	Sample	A/I	N_CH_	N_CA_	N_CO_	Pooled SMD [95 % CI]	p-value
Oxidative damage	8-iso-PGF2α	P or S	A	3	45	101	3.65 [0.26, 7.04]	**0.03**
			I	3	55	101	3.56 [0.23, 6.90]	**0.04**
	AOPP	P or S	A	5	188	143	1.21 [0.57, 1.85]	**<0.001**
			I	3	73	96	0.35 [0.03, 0.67]	**0.03**
	MDA	P or S	A	16	370	527	1.20 [0.70, 1.70]	**<0.001**
			I	14	278	549	0.55 [0.03, 1.08]	**0.04**
Enzymes	PON-1	P or S	A	6	205	232	−1.01 [-1.72, −0.30]	**0.005**
			I	3	71	122	−0.81 [-1.89, 0.27]	0.14
	GPx-EC	P or S	A	8	137	170	0.82 [0.13, 1.50]	**0.02**
			I	8	169	170	0.44 [-0.26, 1.14]	0.22
	GPx-IC	RBC	A	4	141	145	−0.37 [-1.58, 0.83]	0.55
			I	7	208	357	0.05 [-0.52, 0.63]	0.86
	CAT	RBC or WBC	A	4	152	156	−0.75 [-0.98, −0.52]	**<0.001**
			I	6	225	241	−0.21 [-1.11, 0.69]	0.64
	SOD-EC	P or S	A	5	105	111	−0.28 [-0.85, 0.28]	0.32
			I	4	67	81	−0.26 [-0.84, 0.31]	0.37
	SOD-IC	RBC or WBC	A	4	146	145	0.10 [-0.41, 0.60]	0.7
			I	6	224	303	0.64 [-0.42, 1.69]	0.24
Vitamins & carotenoids	β-carotene	P or S	A	6	99	493	−1.52 [-2.08, −0.96]	**<0.001**
			I	8	311	622	−0.69 [-0.91, −0.46]	**<0.001**
	Lycopene	P	A	4	73	468	−1.33 [-1.62, −1.04]	**<0.001**
			I	4	207	468	−0.63 [-0.83, −0.42]	**<0.001**
	Total carotenoids	P	A	3	60	431	−1.29 [-1.71, −0.86]	**<0.001**
			I	3	183	431	−0.66 [-1.17, −0.16]	**0.01**
	Vitamin A	P or S	A	4	58	557	−1.13 [-2.00, −0.26]	**0.01**
			I	6	164	626	−0.55 [-0.78, −0.31]	**<0.001**
	Vitamin C	P or S	A	3	58	92	−1.01 [-1.36, −0.65]	**<0.001**
			I	5	223	221	−0.47 [-0.68, −0.26]	**<0.001**
	β-Cryptoxanthin	P	A	4	73	468	−0.98 [-1.26, −0.70]	**<0.001**
			I	4	207	468	−0.50 [-0.90, −0.11]	**0.01**
	Lutein & zeaxanthin	P	A	4	73	468	−0.82 [-1.26, −0.37]	**<0.001**
			I	4	207	468	0.001 [-0.64, 0.64]	0.999
	α-carotene	P	A	4	73	468	−0.75 [-1.37, −0.14]	**0.02**
			I	4	207	468	−0.41 [-1.06, 0.24]	0.22
	Vitamin E	P or S	A	6	103	612	−0.44 [-1.00, 0.11]	0.12
			I	8	304	741	−0.21 [-0.44, 0.02]	0.07
Proteins	Albumin	P or S	A	23	1297	1222	−1.20 [-1.51, −0.90]	**<0.001**
			I	15	547	662	−0.56 [-0.78, −0.35]	**<0.001**
	SepP	P or S	A	3	50	45	−1.13 [-2.37, 0.10]	0.070
			I	3	68	45	−1.19 [-2.10, −0.28]	**0.01**
	Transferrin	S	A	8	326	273	−0.88 [-1.24, −0.52]	**<0.001**
			I	6	175	223	−0.32 [-0.52, −0.11]	**0.002**
	R–SH	P or S	A	12	470	351	−0.79 [-1.06, −0.53]	**<0.001**
			I	7	221	238	−0.55 [-0.90, −0.21]	**0.002**
Other	TAC	P or S	A	11	371	395	−1.35 [-1.93, −0.77]	**<0.001**
			I	8	260	378	−0.70 [-1.17, −0.24]	**0.003**
	TBIL	S	A	6	750	859	−1.16 [-1.90, −0.43]	**0.002**
			I	5	154	551	−0.56 [-0.83, −0.28]	**<0.001**
	Se	P or S	A	5	112	142	−1.08 [-1.87, −0.29]	**0.008**
			I	8	299	282	−0.65 [-1.01, −0.30]	**<0.001**
	Zn	P or S	A	3	96	90	−0.67 [-1.79, 0.45]	0.24
			I	4	209	184	−0.14 [-0.40, 0.12]	0.28
	SUA	S	A	7	630	598	−0.21 [-0.65, 0.23]	0.35
			I	6	326	428	0.21 [-0.23, 0.65]	0.35

Abbreviations: 8-iso-PGF2α, 8-iso-prostaglandin F2α; A, active disease versus healthy controls; AOPP, advanced oxidation protein products; CAT, catalase; GPx-EC, extracellular glutathione peroxidase; GPx-IC, intracellular glutathione peroxidase; I, inactive disease versus healthy controls; MDA, malondialdehyde; N_CA_, number of patients with IBD (cases) included in the analysis; N_CH_, number of cohorts with IBD included in the analysis; N_CO_, number of healthy controls included in the analysis; P, plasma; Pooled SMD [95 % CI], pooled standardized mean difference with 95 % confidence interval for each analysis; PON-1, Paraoxonase 1; R–SH, plasma free thiols; RBC, red blood cells; S, serum; Se, selenium; SepP, Selenoprotein P; SOD-EC, extracellular superoxide dismutase; SOD-IC, intracellular superoxide dismutase; SUA, serum uric acid; TAC, total antioxidant capacity; TBIL, total bilirubin; WBC, white blood cells; Zn, zinc.

##### 3.3.1.1 Oxidative damage to biomacromolecules

**Fig. 2:**
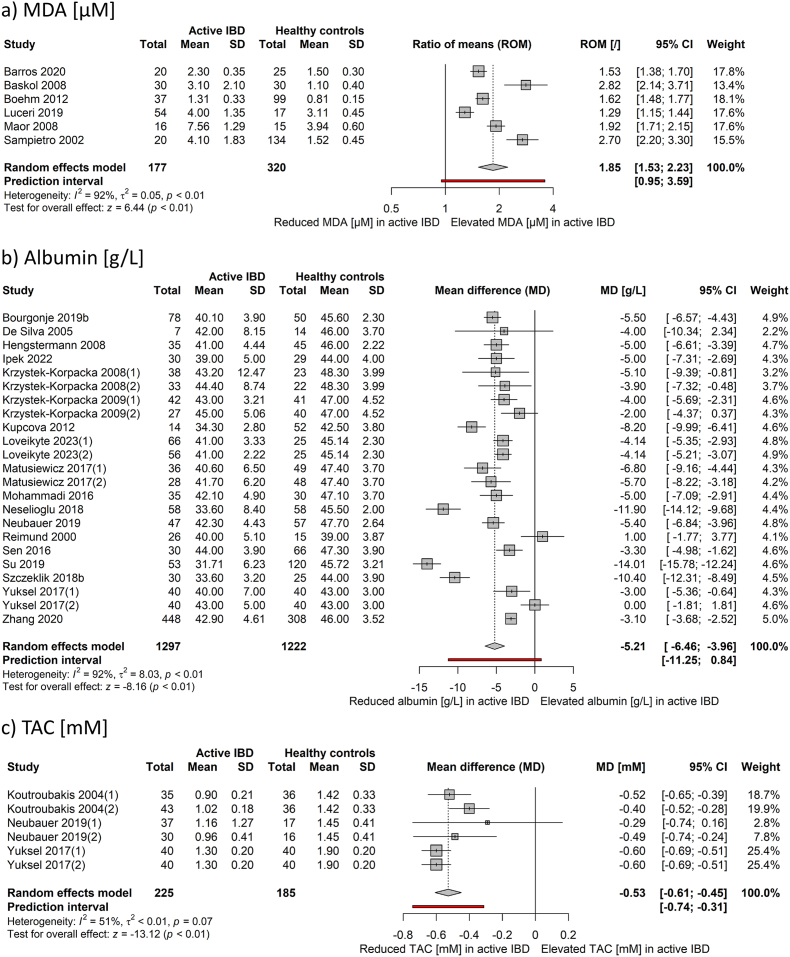
Forest plots of studies examining a) MDA (μM), b) albumin (g/L), and c) TAC (mM) levels in patients with active IBD and healthy controls using MD or ROM as the outcome measures. (1), cohort of patients with Crohn's disease; (2), cohort of patients with ulcerative colitis; CI, confidence interval; IBD, inflammatory bowel disease; MD, mean difference; MDA, malondialdehyde; ROM, ratio of means; SD, standard deviation; TAC, total antioxidant capacity.

Statistically significant large effect sizes were estimated for 8-iso-PGF2α (SMD 3.65, p = 0.03), AOPP (SMD 1.21, p < 0.001), and MDA (SMD 1.20, p < 0.001). All of these markers were elevated in patients with active IBD compared to healthy controls (Table 1). Subsequently, MDA levels from 6 cohorts of patients were subjected to further analysis to obtain the ratio of means of 1.85 (95 % CI: 1.53 to 2.23, p < 0.001) between active IBD and healthy controls as the outcome measure. The underlying 95 % prediction interval was 0.95–3.59 (Fig. 2a). The effect sizes were larger in UC (ratio of means 2.82, 95 % CI: 2.14 to 3.71) than in CD (ratio of means 1.73, 95 % CI: 1.44 to 2.07).

##### 3.3.1.2 Enzymes

Activities of Paraoxonase 1 (SMD −1.01, p = 0.005) and catalase (SMD −0.75, p < 0.001) were significantly reduced in patients with active IBD. GPx activity, measured in plasma or serum, was significantly increased in patients with active IBD (SMD 0.82, p = 0.02). The activities of GPx, measured in erythrocytes, as well as SOD, measured in both plasma and red and white blood cells, showed no significant differences between patients with active IBD and healthy controls (Table 1).

##### 3.3.1.3 Vitamins & carotenoids

Significantly reduced values were found in patients with active IBD compared to healthy controls for β-carotene (SMD −1.52, p < 0.001), lycopene (SMD −1.33, p < 0.001), total carotenoids (SMD −1.29, p < 0.001), vitamin A (SMD −1.13, p = 0.01), vitamin C (SMD −1.01, p < 0.001), β-Cryptoxanthin (SMD −0.98, p < 0.001), lutein and zeaxanthin (SMD −0.82, p < 0.001), and α-carotene (SMD −0.75, p = 0.02). The effect sizes for vitamin E were not significant (Table 1).

##### 3.3.1.4 Proteins

Pooled results showed a significant decrease in levels of albumin (SMD −1.20, p < 0.001), transferrin (SMD −0.88, p < 0.001), and free thiols (SMD −0.79, p < 0.001) in patients with active IBD when compared to the healthy control group. Levels of Selenoprotein P did not differ between the two groups (Table 1). A detailed analysis was carried out for albumin using mean difference as the outcome measure (Fig. 2b). A total of 23 cohorts of patients with IBD were included in the analysis. The estimated average mean difference based on the random-effects model was −5.21 g/l (95 % CI: −6.46 to −3.96, p < 0.001) with a 95 % prediction interval of −11.25 to 0.84 g/l. A subgroup analysis revealed that both patients with active CD and active UC had, on average, lower albumin concentration compared to healthy controls, with reductions of −5.82 g/l (95 % CI: −8.08 to −3. 55 g/l, p < 0.001) for CD and −4.41 g/l (95 % CI: −6.30 to −2.52 g/l, p < 0.001), for UC, respectively.

##### 3.3.1.5 Other

Large effect sizes were calculated for TAC (SMD −1.35, p < 0.001), TBIL (SMD −1.16, p = 0.002), and selenium levels (SMD −1.08, p = 0.008), all of which exhibited significant reductions in individuals with active IBD compared to healthy controls. No significant differences were observed for SUA and zinc levels (Table 1). TAC was further analyzed based on 6 cohorts of patients from similar analytical studies (Fig. 2c). The average mean difference between the active IBD and healthy controls across studies was −0.53 mmol/l (95 % CI: −0.61 to −0.45 mmol/l, p < 0.001), with similar effect sizes for active CD (−0.56 mmol/l, 95 % CI: −0.65 to −0.47 mmol/l, p < 0.001) and active UC (−0.50 mmol/l, 95 % CI: −0.65 to −0.35 mmol/l, p < 0.001). The 95 % prediction interval for active IBD was −0.74 to −0.31 mmol/l.

#### 3.3.2 Inactive IBD versus healthy controls

Levels of 27 oxidative stress-related biomarkers were evaluated in patients with inactive IBD and healthy controls across 40 studies, with the majority of studies evaluating more than one biomarker. In addition to intracellular GPx, catalase, and SOD, other biomarkers were determined in plasma or serum. The sample size of inactive patients included in the meta-analysis ranged from 55 (8-iso-PGF2α) to 547 (albumin), and from 45 (Selenoprotein P) to 741 (vitamin E) for healthy controls. Detailed results of the meta-analysis are provided in Tables 1 and in Supplementary Fig. 1.

##### 3.3.2.1 Oxidative damage to biomacromolecules

All three markers of oxidative damage were significantly increased in patients with inactive IBD as compared to the healthy controls. 8-iso-PGF2α accumulated the most (SMD 3.56, p = 0.04), followed by MDA (SMD 0.55, p = 0.04), and AOPP (SMD 0.35, p = 0.03) (Table 1).

##### 3.3.2.2 Enzymes

There were no significant differences observed between patients with inactive disease and healthy controls in terms of the activities of any of the evaluated enzymes, which included Paraoxonase 1, GPx, catalase, and SOD (Table 1).

##### 3.3.2.3 Vitamins & carotenoids

Patients with inactive IBD had significantly lower levels of β-carotene (SMD −0.69, p < 0.001), total carotenoids (SMD −0.66, p = 0.01), lycopene (SMD −0.63, p < 0.001), vitamin A (SMD −0.55, p < 0.001), β-Cryptoxanthin (SMD −0.50, p = 0.01), and vitamin C (SMD −0.47, p < 0.001) when compared to healthy individuals. No significant differences were observed between the two groups for lutein and zeaxanthin, α-carotene, and vitamin E (Table 1).

##### 3.3.2.4 Proteins

Significantly lower levels of all evaluated biomarkers were observed in patients with inactive IBD as compared to healthy controls, including Selenoprotein P (SMD −1.19, p = 0.01), albumin (SMD −0.56, p < 0.001), R–SH (SMD −0.55, p = 0.002), and transferrin (SMD −0.32, p = 0.002) (Table 1).

##### 3.3.2.5 Other

Compared with healthy controls, levels of the following markers were significantly decreased in patients with inactive IBD: TAC (SMD −0.70, p = 0.003), selenium (SMD −0.65, p < 0.001), and TBIL (SMD −0.56, p < 0.001). No significant differences were observed for concentrations of zinc and SUA (Table 1).

#### 3.3.3 Active Crohn's disease and ulcerative colitis versus respective inactive controls

**Fig. 3:**
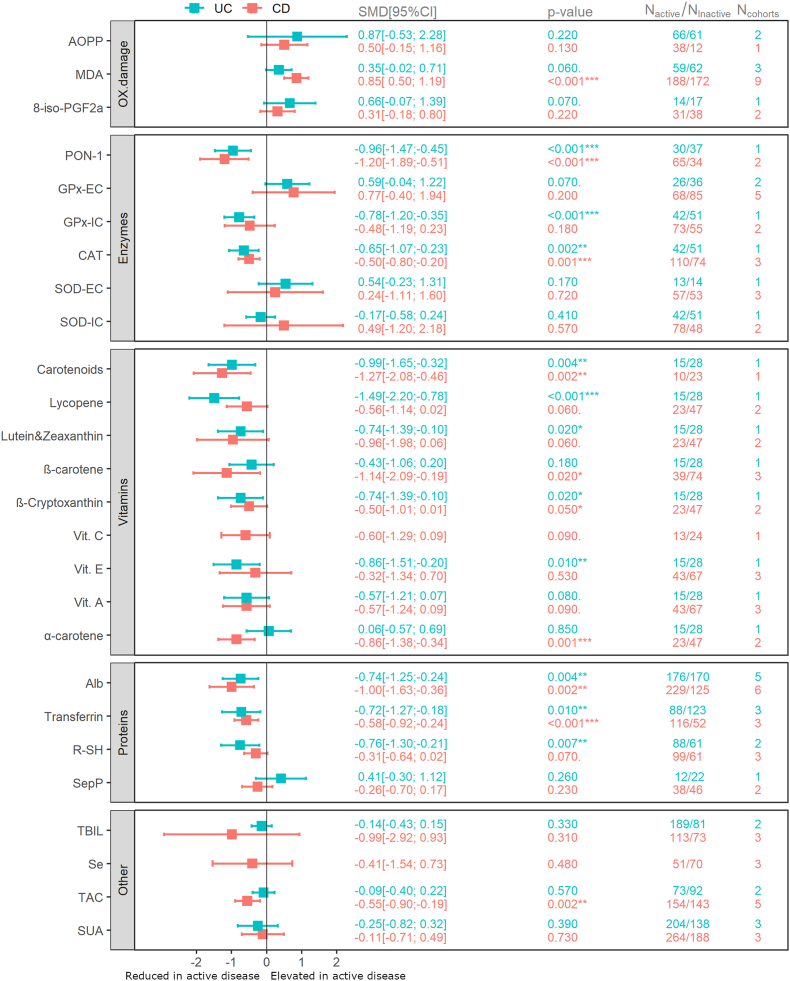
Oxidative stress-related biomarkers in active CD and UC versus their respective inactive controls. 8-iso-PGF2α, 8-iso-prostaglandin F2α; Alb, albumin; AOPP, advanced oxidation protein products; CAT, catalase; CD, Crohn's disease; GPx-EC, extracellular glutathione peroxidase measured in plasma; GPx-IC, intracellular glutathione peroxidase measured in red blood cells; MDA, malondialdehyde; N_active_, number of patients with active disease included in the analysis; N_cohorts_, number of cohorts of patients with active disease included in the analysis; N_inactive_, number of patients with inactive disease included in the analysis; OX. damage, oxidative damage to biomacromolecules; SMD [95 % CI], pooled standardized mean difference with 95 % confidence interval for each analysis; PON-1, Paraoxonase 1; R–SH, plasma free thiols; Se, selenium; SepP, Selenoprotein P; SOD-EC, extracellular superoxide dismutase measured in plasma; SOD-IC., intracellular superoxide dismutase; SUA, serum uric acid; TAC, total antioxidant capacity; TBIL, total bilirubin; UC, ulcerative colitis; Vit. A, vitamin A; Vit. C, vitamin C; Vit. E, vitamin E. (For interpretation of the references to colour in this figure legend, the reader is referred to the Web version of this article.)

A subgroup analysis of 24 biomarkers was conducted to explore potential distinctions between active and inactive disease states of either CD or UC, as well as the differences between the two IBD subtypes themselves. Data for two additional biomarkers, specifically vitamin C and selenium, were limited to CD. The analysis included patients ranging from 10 to 264 for active CD and 12 to 188 for inactive controls. Similarly, there were between 12 and 204 patients with active and between 14 and 170 with inactive UC included in the analysis of individual biomarkers. Further details are provided in Fig. 3 and in the Supplementary Fig. 2.

##### 3.3.3.1 Oxidative damage to biomacromolecules

Concentrations of MDA were significantly elevated in patients with active CD compared to the inactive CD (SMD 0.85, p < 0.001), while the differences observed for the patients with UC were not statistically significant. There were no differences in AOPP and 8-iso-PGF2α levels between patients with active and inactive disease, regardless of the IBD subtype (Fig. 3).

##### 3.3.3.2 Enzymes

Enzymatic activities were significantly reduced in patients with active compared to those with inactive disease for the following enzymes: Paraoxonase 1 in both CD (SMD −1.20, p < 0.001) and UC (SMD −0.96, p < 0.001), GPx measured in erythrocytes of patients with UC (SMD −0.78, p < 0.001), and catalase in both CD (SMD −0.50, p = 0.001) and UC (SMD −0.65, p = 0.002) (Fig. 3).

##### 3.3.3.3 Vitamins & carotenoids

Levels of the following markers were significantly reduced in patients with active compared to inactive disease: total carotenoids in CD (SMD −1.27, p = 0.002) and UC (SMD −0.99, p = 0.004), lycopene in UC (SMD −1.49, p < 0.001), lutein and zeaxanthin in UC (SMD −0.74, p = 0.02), β-carotene in CD (SMD −1.14, p = 0.02), β-Cryptoxanthin in UC (SMD −0.74, p = 0.02), vitamin E in UC (SMD −0.86, p = 0.01), and α-carotene in CD (SMD −0.86, p = 0.001) (Fig. 3). The differences in calculated effect sizes between UC and CD were significant for lycopene (p = 0.05, Supplementary Fig. 2k) and α-carotene (p = 0.03, Supplementary Fig. 2q).

##### 3.3.3.4 Proteins

Pooled data showed significantly lower levels of albumin in patients with active CD (SMD −1.00, p = 0.002) and UC (SMD −0.74, p = 0.004) compared to those with inactive disease. Similarly, levels of transferrin were significantly lower in both patients with active UC (SMD −0.72, p = 0.01) and CD (SMD −0.58, p < 0.001) when compared to those with inactive disease. Additionally, R–SH were significantly reduced in patients with active UC in comparison to patients with inactive UC (SMD −0.76, p = 0.007) (Fig. 3).

##### 3.3.3.5 Other

Significantly lower TAC values were observed in patients with active CD compared to controls with inactive CD (SMD −0.55, p = 0.002) (Fig. 3).

## 4 Discussion

**Fig. 4:**
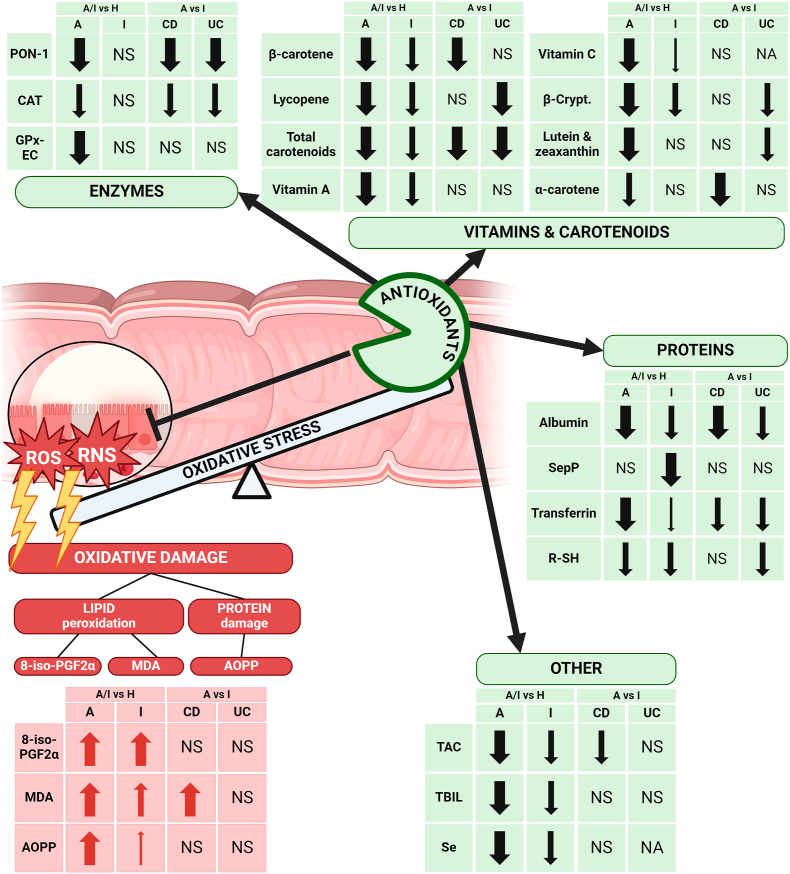
Overview of oxidative stress-related biomarkers in patients with IBD. Markers of oxidative damage to lipids (8-iso-PGF2α, MDA) and proteins (AOPP) are increased, while antioxidant levels, including various enzymes, vitamins, carotenoids, proteins, and other antioxidants are generally reduced in IBD patients. Significant reductions (↓), increases (↑), non-significant changes (NS), and data not available (NA) are shown for both Crohn's disease (CD) and ulcerative colitis (UC) in comparisons between active disease (A), inactive disease (I) and healthy controls (H). The width of the arrows reflects the effect size as standardized mean difference (SMD), with narrow arrows indicating a small (SMD <0.5), medium arrows representing moderate (0.5 ≤ SMD <0.8), and wide arrows indicating large effect size (SMD ≥0.8). 8-iso-PGF2α, 8-iso-prostaglandin F2α; AOPP, advanced oxidation protein products; β-Crypt., β-cryptoxanthin; CAT, catalase; CD, Crohn's disease; GPx-EC, extracellular glutathione peroxidase measured in plasma; MDA, malondialdehyde; PON-1, Paraoxonase 1; RNS, reactive nitrogen species; ROS, reactive oxygen species; R–SH, plasma free thiols; Se, selenium; SepP, Selenoprotein P; TAC, total antioxidant capacity; TBIL, total bilirubin; UC, ulcerative colitis.

Previous studies have systematically reviewed the existing literature on oxidative stress-related biomarkers in IBD [16]. However, to the best of our knowledge, this study is the first to not only identify but also quantitatively evaluate all published oxidative stress-related biomarkers in individuals with IBD and healthy controls. The included studies were heterogeneous since they included patients with either CD or UC, and in some cases, the IBD subtype was not specified (Supplementary Table 2). Similarly, various activity parameters were used to classify the disease as either active or quiescent and this lack of standardization makes comparisons between studies less reliable (Supplementary Table 2). Moreover, most of the evaluated markers are not routinely measured in laboratories, and variations in the analytical methods were observed across studies. To address these discrepancies, SMD was employed as a measure of effect size, enabling us to compare differences in the levels of investigated biomarkers between patients with active IBD and healthy controls as our primary objective. Furthermore, the use of SMD allowed for a standardization of these differences onto a uniform scale. This facilitated quantitative comparisons between otherwise disparate biomarkers, providing insight into which biomarkers demonstrate the strongest associations with disease activity. Such information holds potential value in identifying surrogate biomarkers of underlying inflammation in patients with IBD. Fig. 4 provides an overview of oxidative stress-related dysregulations in patients with IBD.

### 4.1 Oxidative damage to biomacromolecules

Biomacromolecules such as lipids and proteins are susceptible to oxidative damage caused by reactive oxygen species. Excess un-neutralized hydrogen peroxide, produced in inflamed intestinal epithelial cells, is converted into highly damaging hydroxyl radicals, which can cause extensive oxidative damage to structures essential for preserving the integrity of the colonic epithelial barrier [84]. Together with hydroperoxyl radicals, these reactive species attack double carbon-carbon bonds in lipids, causing lipid peroxidation and subsequent damage to cells, tissues, and organs [85]. Once the process begins, it progresses through the propagation phase and continues until the final lipid peroxidation products are formed, unless interrupted by antioxidants. Polyunsaturated fatty acids, such as arachidonic acid, are key substrates for lipid peroxidation, resulting in the formation of end-products like MDA and 8-iso-PGF2α [85]. Both biomarkers were significantly elevated in active and inactive IBD compared to healthy controls, indicating oxidative damage to lipids in IBD.

The largest effect size was computed for plasma 8-iso-PGF2α (Table 1). However, it is essential to note that these results are less reliable, given that analysis included only two studies [63,78] with a total of 45 patients with active and 55 with inactive IBD. While 8-iso-PGF2α is stable and present in relatively large amounts in biological fluids, specific to lipid peroxidation, and synthesized *in vivo* [63], further research is needed to explore its potential role as a plasma biomarker of IBD activity.

Contrary to 8-iso-PGF2α, MDA has been extensively studied in patients with IBD, providing further evidence of lipid peroxidation. According to our MDA findings, it is possible to differentiate not only between healthy individuals and those with IBD (Table 1) but also between patients with active and inactive Crohn's disease (Fig. 3). Since SMD values lack clinical interpretability, our focus was on establishing plasma MDA cut-off values for active IBD by further analysing 6 studies [28,39,41,44,58,68] employing similar spectrophotometric thiobarbituric acid-based assays (Fig. 2a). However, it is worth mentioning that MDA levels displayed high variability even within the healthy control groups of included studies (range: 0.81–3.94 μM) (Fig. 2a). In complex biological systems many compounds react with thiobarbituric acid, potentially contributing to the observed variations [86]. Additionally, Boehm et al. modified the original analytical method for measuring the derivates of thiobarbituric acid [44]. Moreover, authors used different biological samples, with four studies utilizing plasma [39,41,44,68] and two employing serum [28,58]. Finally, studies show that levels of MDA are also sensitive to diurnal variations and exercise [87,88]. Taking all factors into account, determining accurate and reliable cut-off values for MDA in active IBD posed a significant challenge. Therefore, we calculated the ratio of means of 1.85 (95 % CI 1.53–2.23) (Fig. 2a). This indicates that, on average, MDA levels in plasma are 1.85 times higher in patients with active IBD compared to healthy individuals, suggesting the involvement of oxidative stress in IBD.

Witko-Sarsat et al. suggested that, instead of lipid peroxidation markers, markers of oxidative damage to proteins, namely AOPP, are more accurate markers of oxidative stress [89]. Human myeloperoxidase, released from activated neutrophils, monocytes, and tissue macrophages, converts the hydrogen peroxide in the presence of chloride ions into the potent oxidizing hypochlorous acid to protect against bacteria [90]. However, this process also leads to the formation and accumulation of AOPP, which are dityrosine-containing and cross-linked products, formed by the reaction of plasma proteins, mainly albumin, with chlorinated compounds [91]. Most protein damage is nonrepairable and can lead to protein fragmentation, aggregation, and loss of function [90]. AOPP were first evaluated in IBD in 2008 by Baskol et al. [41] and Krzystek-Korpacka et al. [54], which explains the limited availability of relevant studies. Nevertheless, preliminary results are promising, as our findings indicate AOPP's potential to distinguish between patients with active IBD and healthy controls, as well as between patients with inactive disease and healthy individuals (Table 1). Moreover, Hamouda et al. [92] suggested that AOPP could help in the early detection of UC-associated mucosal dysplasia, as significantly higher levels were observed in patients with dysplasia than in those without dysplasia.

While our search identified additional markers of oxidative damage to biomacromolecules, namely protein carbonyls [28], nitro-tyrosine [93], chlorinated tyrosine [94], and ischaemia-modified albumin [51], the limited number of relevant studies prevented their inclusion in the meta-analysis.

In summary, our findings suggest that 8-iso-PGF2α, MDA, and AOPP have the potential to distinguish patients with active and inactive IBD from healthy individuals, with MDA demonstrating the additional capability to detect exacerbations in CD.

### 4.2 Enzymes

To prevent oxidative damage, the human body primarily relies on enzymatic antioxidants such as Paraoxonase 1, catalase, superoxide dismutase, and GPx, which neutralize reactive species and prevent the formation of new ones [95]. In contrast to the data on oxidative damage, studies assessing enzymatic antioxidant activities in IBD are less concise and exhibit varying degrees of agreement. Our results demonstrate significant differences between patients with active IBD and the healthy population in terms of plasma Paraoxonase 1, erythrocyte and immune cell catalase, and plasma GPx levels. Specifically, the first two are decreased, while the latter is increased (Table 1). However, patients with inactive disease did not display any significant differences from healthy controls in relation to the assessed antioxidant enzymes (Table 1). Our findings also indicate a significant decrease in Paraoxonase 1 levels in active cases of UC and CD when compared to their respective inactive states (Fig. 3).

Paraoxonase 1 is known as an antioxidant enzyme because of its ability to hydrolyse lipid peroxides and counteract oxidative stress [96]. The observed decrease in the enzyme's activity may, therefore, be attributed to the saturation of its natural capacity to protect against lipid peroxidation. Additionally, Paraoxonase 1 is inactivated by oxidized low-density lipoproteins and its expression is downregulated by proinflammatory cytokines, specifically interleukin-1 and tumor necrosis factor α [97,98]. In line with this, Szczeklik et al. [73] identified a very strong negative correlation between Paraoxonase 1 and MDA in patients with CD, and Baskol et al. [40] measured lower Paraoxonase 1 activity and higher MDA concentrations in patients with UC compared to healthy controls.

Similar to Paraoxonase 1, though with a smaller estimated effect size, catalase measured in red and white blood cells exhibited the potential to distinguish between active IBD and healthy controls (Table 1), as well as between active UC and CD in contrast to their respective inactive states (Fig. 3). Catalase plays a pivotal role in neutralizing hydrogen peroxide, a precursor of hydroxyl radicals that, in turn, cause lipid peroxidation. Decreased catalase activity is an indicator of insufficient antioxidant defence mechanisms, possibly resulting from enzyme damage or exhaustion of catalase processing capacity due to elevated substrate levels during oxidative stress [27,56]. Krzystek-Korpacka et al. [56] observed a negative correlation between catalase activity and both Crohn's Disease Activity Index and C-reactive protein in patients with CD. Additionally, they reported a negative correlation between endoscopic activity of the disease and catalase in patients with UC. Furthermore, markers of oxidative damage, specifically MDA [27] and AOPP [56], displayed a negative correlation with catalase activity in patients with CD. This observation provides additional support for catalase's role as an oxidative stress as well as disease activity biomarker.

Finally, included studies investigating SOD and GPx levels, whether in erythrocytes, white blood cells or plasma, reported conflicting results (Table 1, Fig. 3, Supplementary Figs. 1e, 1f, 1h, and 1i). Superoxide dismutase, a vital antioxidant enzyme in aerobic cells, converts superoxide radicals into oxygen and hydrogen peroxide. The hydrogen peroxide is further reduced to water by GPx, which also detoxifies lipid peroxides, thereby preventing lipid peroxidation [95]. The inconclusive results regarding these enzymes could be due to several factors, including the duration of the disease, disease activity, the timing of sample collection, and the percentage of smokers, which collectively influence the overall results [72,78]. Furthermore, adaptive homeostatic responses to oxidative stress can also impact the levels of these enzymes [39]. We did observe a significant difference between patients with active and inactive UC in terms of erythrocyte GPx, however, it is important to note that our analysis relied on data from only one study (Fig. 3). Our findings also suggest an increase in plasma GPx levels among patients with active IBD compared to healthy controls (Table 1), however, results of studies measuring GPx and SOD in plasma should be approached with caution. These enzymes are primarily intracellular, and their plasma activities are several orders of magnitude lower than their levels in erythrocytes, making their measurements in plasma less reliable [16,56].

Based on our preliminary findings, catalase measured in red and white blood cells, along with Paraoxonase 1 measured in plasma, emerged as the most promising candidates among enzymatic antioxidants to distinguish patients with active IBD from healthy controls. Additionally, these markers show potential in detecting exacerbation in both CD and UC.

### 4.3 Vitamins & carotenoids

Among the various markers analyzed, carotenoids, particularly α-carotene, β-carotene, β-cryptoxanthin, lycopene, lutein, and zeaxanthin, exhibited the most consistent findings and were found to be unanimously decreased in patients with active IBD compared to healthy controls (Table 1). These compounds are efficient non-enzymatic antioxidants that quench the singlet molecular oxygen and deactivate peroxyl radicals by scavenging them and forming resonance-stabilized carbon-centered radical adducts, thereby preventing oxidative damage to cellular membranes and lipoproteins [95,99]. In conditions of chronic inflammation like IBD, there is an increased demand for antioxidants [50], potentially leading to the depletion of carotenoid levels. This aligns with our findings of more pronounced depletion in active disease when compared to periods of disease inactivity (Fig. 3). Furthermore, carotenoids rank among the most prevalent lipid-soluble phytonutrients, naturally occurring in a wide array of fruits and vegetables [95]. Consequently, malnutrition, different eating habits, dietary restrictions, and malabsorption, which are frequently encountered in patients with IBD [26,50], can further contribute to lower levels of carotenoids. As highlighted by Hengstermann et al. [50], dietary recommendations promoting increased intake of fruits, vegetables, and even supplements may be beneficial for patients with IBD. It is noteworthy that we observed differences in the levels of lycopene and α-carotene between CD and UC (Fig. 3, Supplementary Figs. 2k and 2q); however, the available data is limited, and further research is needed to elucidate their potential as diagnostic markers.

Our study also observed reduced levels of vitamins C and A in both active and inactive IBD (Table 1). Vitamin C, a water-soluble antioxidant, and vitamin A, a fat-soluble antioxidant, both act as free radical scavengers and help prevent lipid peroxidation [95]. The reduced levels of these dietary antioxidants may be due to factors similar to those affecting carotenoids, including increased demand due to active inflammation and oxidative stress, malabsorption, and reduced intake of fruits and vegetables [100]. Additionally, proinflammatory tumor necrosis factor α has been shown to impair vitamin C uptake [101]. Furthermore, reduced levels of carotenoids such as β-carotene, β-cryptoxanthin, and α-carotene, which are precursors to vitamin A [102], can contribute to reduced vitamin A levels in IBD patients.

While vitamins and carotenoids appear promising as surrogate biomarkers of IBD activity, their measurements in clinical practice may pose challenges due to the lack of standardization with respect to variations in food intake.

### 4.4 Proteins

Within the scope of the analyzed protein biomarkers, albumin, transferrin, and R–SH emerged as particularly promising indicators of disease activity, exhibiting significant decreases in levels among individuals with IBD compared to healthy controls. This reduction was more pronounced in active disease states than in periods of disease inactivity (Table 1). Furthermore, all these biomarkers exhibit potential in detecting disease exacerbations, allowing for differentiation between patients with active and inactive UC, and, for albumin and transferrin, between patients with active and inactive CD as well (Fig. 3). Data on Selenoprotein P are less conclusive and based on only two studies (Table 1, Fig. 3).

Albumin, a routinely measured biomarker in clinical practice, stood out as the most frequently assessed among the 27 biomarkers included in our analysis. It belongs to the group of negative acute-phase reactants, and decreased levels may be observed during inflammation. However, conditions such as malnutrition and malabsorption can also contribute to low albumin levels [14]. Additionally, under conditions of oxidative stress, albumin serves as the primary substrate for the formation of AOPP [91]. This is in line with our findings, as we observed lower albumin levels and accumulation of AOPP in patients with active IBD when compared to patients with inactive disease and healthy controls (Table 1). On average, serum albumin levels in patients with active IBD were 5.21 g/l lower than those in healthy individuals (Fig. 2b). These results are consistent with other studies that have confirmed negative correlations between disease activity and albumin levels in CD [59,74] and UC [59]. Moreover, hypoalbuminemia is included in the clinical practice guidelines as one of the laboratory markers that can predict the need for colectomy in acute severe UC [103].

Human serum albumin is also the most abundant thiol in plasma, mostly present in reduced form [104]. Together with low molecular weight reduced thiols, such as glutathione and cysteine and its derivates, it forms the pool of R–SH, which are one of the main plasma antioxidants. In oxidative stress, thiol oxidation products are formed and R–SH levels are diminished [104]. This, combined with the previously discussed hypoalbuminemia, contributes to the observed lower R–SH levels in our analysis, emphasizing the potential of R–SH as a surrogate marker for assessing IBD activity. Moreover, Bourgonje et al. discovered that R–SH are superior to fecal calprotectin in reflecting endoscopic disease activity in IBD [23].

Like albumin, transferrin is a negative acute phase reactant, and its levels decrease in response to inflammation [14]. Transferrin plays a crucial role in reducing oxidative stress by binding free iron in circulation and tissues. This prevents free iron from catalyzing the conversion of hydrogen peroxide into highly reactive hydroxyl radicals through the Fenton reaction [105]. Therefore, reduced transferrin levels during inflammation can exacerbate oxidative stress, which may help explain the increased oxidative stress observed in patients with active IBD (Table 1, Fig. 3). In accordance, Matusiewicz et al. [59] observed a positive correlation between transferrin levels and albumin, as well as a negative correlation with both Crohn's Disease Activity Index and Ulcerative Colitis Activity Index.

According to our results, albumin and transferrin show potential as surrogate markers of disease activity in both subtypes of IBD. Among the two, albumin has been more extensively studied and the certainty of evidence is higher.

### 4.5 Other

The remaining oxidative stress-related biomarkers could not be categorized into any of the previously discussed groups and were consequently grouped together under the label 'other'. TAC, TBIL and selenium levels exhibited significant differences between patients with active IBD and healthy controls, as well as between those with inactive disease and healthy individuals (Table 1). Moreover, based on TAC levels, we could detect exacerbations of CD (Fig. 3).

Rather than individual components, TAC considers the cumulative effect of all antioxidants present in plasma, including proteins, enzymes, vitamins and carotenoids, and other low molecular weight antioxidants. Therefore, TAC may provide more relevant biological information than measurements of any of the individual components [106]. In chronic inflammatory conditions, persistent depletion of antioxidant reserves in circulating fluids, coupled with variations in dietary intake, malabsorption, elevated vitamin requirements, and heightened gastrointestinal losses, collectively lead to a decrease in TAC [53]. In accordance, our results indicate that, on average, TAC is reduced by 0.53 mmol/l Trolox equivalents in patients with active IBD compared to healthy controls (Fig. 2c).

TBIL, which is also indirectly measured by TAC, is an important endogenous plasma antioxidant, effectively scavenging peroxyl radicals [104,107]. Our analysis revealed a consistent trend among studies, indicating reduced TBIL levels in active IBD cases (Table 1). Additionally, Szczeklik et al. [74] observed a significant negative correlation between TBIL and Crohn's Disease Activity Index. Inflammation-mediated increased oxidative stress, rather than genetic predisposition, is likely the primary driver of increased consumption of antioxidant TBIL in IBD, occurring even before the disease's clinical manifestation [108]. Among the included studies, Sen et al. [69] remains unique in its findings, documenting an elevation in patients with active and a reduction in patients with inactive CD, attributing it to oxidative stress, yet failing to establish a correlation with Crohn's Disease Activity Index. Moreover, Tian et al. suggested that the quality of evidence from the study by Sen et al. might be limited due to the small sample size [75]. By excluding the study by Sen et al., we obtained one of the largest SMDs (−1.50, p < 0.001) among all the evaluated markers for active IBD versus healthy controls (Table 1). Moreover, we were then able to detect differences in the biomarker levels between UC and CD, previously calculated only for lycopene and α-carotene (Fig. 3). This highlights the necessity for further research to explore TBIL's potential as a surrogate marker of IBD activity, especially given its advantage as a standardized biochemical parameter routinely measured in clinical practice.

Selenium is an essential micronutrient, incorporated as selenocysteine at the active site of a wide range of selenoproteins with antioxidant activities, including GPx and Selenoprotein P [109]. Therefore, selenium deficiency may increase susceptibility to oxidative damage. Barros et al. [39] demonstrated a 5.1-fold increase in lipid peroxidation in patients with CD associated with plasma selenium deficiency. Reduced intake of selenium-rich foods, impaired absorption, enhanced antioxidant consumption due to ongoing inflammation and transcapillary escape of selenium proteins due to endothelial injury are all contributing factors to lower selenium levels in patients with IBD [39]. While our study yielded promising results (Table 1), the reliability of selenium as a potential biomarker of IBD activity remains uncertain due to the lack of food intake standardisation.

SUA possesses strong radical scavenging activity and is an important and routinely measured plasma antioxidant [95,104]. However, we were unable to detect differences in SUA levels between the investigated patient groups and healthy controls (Table 1). Included studies reported contradictory results, with some observing a decrease in SUA levels in patients with active IBD [62,71], while others noted an increase [81] (Supplementary Fig. 1a). Zhu et al. [81], the only authors to observe an increase in SUA levels, standardized their results to creatinine levels, a practice not employed in other studies. Since creatinine levels themselves might vary across the groups, the significance of SUA as a biomarker of disease activity becomes less apparent. In a sensitivity analysis, after excluding the study by Zhu et al. [81], SUA was significantly (SMD -0.53, p = 0.005) reduced in patients with active IBD compared to healthy controls. Therefore, SUA might still have a potential as a biomarker in IBD; however, further research is needed.

TAC and TBIL show the greatest potential as biomarkers of disease activity in IBD, however, the potential of SUA must not be overlooked.

### 4.6 Oxidative stress in inactive disease

We observed persistent oxidative stress-related dysregulations in patients with IBD who were classified as inactive under the current guidelines, giving insight into the underlying pathogenic mechanisms. Biomarkers of oxidative damage to biomacromolecules (8-iso-PGF2α, AOPP, MDA) were significantly elevated, while vitamins & carotenoids (β-carotene, lycopene, total carotenoids, vitamin A, vitamin C, β-Cryptoxanthin), proteins (albumin, Selenoprotein P, transferrin, R–SH), and other markers (TAC, TBIL, selenium) were significantly reduced in inactive disease compared to healthy individuals. Similarly, studies by Siebert et al. [110], and Arijs et al. [10] identified ongoing cellular, molecular, and microbial activity, as well as immune-related gene expression abnormalities in patients with endoscopically inactive IBD and suggested targets for future therapies. A novel treatment objective under consideration in IBD is molecular healing, involving the restoration of specific molecular pathways implicated in the disease's etiopathogenesis [9,11]. The identified oxidative stress-related biomarkers may thus serve as potential indicators, not only of the depth of remission but also as predictors of disease flares. Integrating them into the concept of disease clearance could represent an ambitious treatment goal in clinical practice. Furthermore, studies have demonstrated an increased risk of cardiovascular disease in patients with IBD [111]. Therefore, identifying individuals with dysregulated oxidative stress-related biomarkers could guide patient management aimed at reducing cardiovascular risk.

### 4.7 Limitations

The present study has some limitations that need to be addressed. We focused on studies published from 2000 onward, motivated by the relatively recent emergence of oxidative stress as a concept in IBD and the inclusion of modern analytical methods. Furthermore, the certainty of evidence in our study varied among biomarkers, influenced by factors such as the number of studies and patients included, as well as the handling of missing data (e.g. standard deviation). The omission of a risk of bias assessment in this study can be attributed to the rigorous implementation of strict inclusion and exclusion criteria during the selection of studies. These criteria likely ensured the inclusion of studies with high methodological quality and reduced the likelihood of bias.

However, in the scope of methodological quality of the included studies, we have observed some drawbacks. Firstly, the diverse set of parameters was used to assess IBD activity, encompassing endoscopic, biochemical, histological, and clinical measures (Supplementary Table 2). Our reliance on the authors' definition of disease activity may lead to the inclusion of a heterogeneous group of patients within either the active or inactive disease classification. Secondly, given the observational nature of the included studies, controlling for external factors that influence plasma concentrations of oxidative stress and inflammation markers (e. g. diet, exercise, co-medication, co-morbidities) is challenging. Thirdly, a lack of standardization in the timing of sampling in the included studies poses another limitation, as some oxidative stress-related biomarkers are susceptible to day-to-day and diurnal variability [112]. Distinguishing changes in biomarker levels from normal variations is challenging under these circumstances. Ideally, sampling should be performed at fixed times across studies to provide a better insight. Under extremely controlled environments, we would anticipate a decrease in variability; however, the applicability of biomarkers in clinical practice would be limited. Fourthly, the analytical variability across included studies presents a challenge. Among the 27 evaluated biomarkers, only a few are routinely measured in biochemical laboratories (e.g. albumin, TBIL, SUA). To successfully determine the utility of an individual biomarker, establish a reference range and implement it in clinical practice, a standardization in analytical methods is required. Finally, the majority of results are derived from groups of unrelated patients, one representing active disease and another inactive disease. To minimize the inter-individual variability, future studies should ideally be longitudinal and include a single group of patients, measuring biomarkers during both the active and inactive periods of IBD. Additionally, healthy control groups should be as similar as possible, matching at least for age and sex.

Despite these limitations, our study provides valuable insights into oxidative stress-related biomarkers in IBD, their associations with disease activity, and lays the groundwork for their potential utilization in disease management.

## 5 Conclusions

This is the first comprehensive meta-analysis to evaluate oxidative stress-related biomarkers in IBD and their associations with disease activity. The observed accumulation of markers of oxidative damage to biomacromolecules, coupled with concurrent reductions in various antioxidants in both patients with active and inactive disease, strongly implies the involvement of oxidative stress in the pathogenesis of IBD. Additionally, our study identified oxidative stress-related biomarkers capable of distinguishing between active and inactive CD. These include biomarkers of oxidative damage to biomacromolecules (MDA), enzymes (Paraoxonase 1, catalase), proteins (albumin, transferrin), and other markers (TAC). Similarly, the following biomarkers show promise in detecting UC exacerbations: enzymes (Paraoxonase 1, erythrocyte GPx, catalase), and proteins (albumin, transferrin, R–SH). Furthermore, vitamins and carotenoids emerged as potential biomarkers of CD and UC activity; however, their intake should be monitored to obtain meaningful results. Oxidative stress-related biomarkers hold potential as minimally invasive tools for disease monitoring and detecting IBD relapse, aimed at achieving long-term treatment goals in IBD.

## CRediT authorship contribution statement

**Armando Tratenšek:** Writing – original draft, Validation, Software, Formal analysis, Data curation, Conceptualization. **Igor Locatelli:** Writing – review & editing, Visualization, Validation, Software, Methodology. **Iztok Grabnar:** Writing – review & editing. **David Drobne:** Writing – review & editing, Conceptualization. **Tomaž Vovk:** Writing – review & editing, Visualization, Validation, Supervision, Conceptualization.

## Declaration of competing interest

The authors declare that they have no known competing financial interests or personal relationships that could have appeared to influence the work reported in this paper.

## Data Availability

Data will be made available on request.
