# Supplementary material for: Oxidative stress-related biomarkers as promising indicators of inflammatory bowel disease activity: A systematic review and meta-analysis
Source: Redox Biol. 2024 Oct 1;77:103380. doi: 10.1016/j.redox.2024.103380 (PMC11490685; doi:10.1016/j.redox.2024.103380)
Supplement: Multimedia component 1 [file mmc1.docx]

**Supplementary table 1.** Detailed search strategies for Ovid MEDLINE, Ovid Embase, and Web of Science.

**Ovid MEDLINE, Ovid Embase**

**Database(s): Embase (**1974 to 2024 May 10) and **Ovid MEDLINE(R) and In-Process & Other Non-Indexed Citations and Daily** (1946 to May 10, 2024)

Search Strategy:

| **#** | **Searches** |
| --- | --- |
| 1 | oxidative stress.mp. [mp=ti, bt, ab, ot, nm, hw, fx, kf, ox, px, rx, ui, an, sy, tn, dm, mf, dv, dq] |
| 2 | oxidative damage.mp. [mp=ti, bt, ab, ot, nm, hw, fx, kf, ox, px, rx, ui, an, sy, tn, dm, mf, dv, dq] |
| 3 | antioxida*.mp. [mp=ti, bt, ab, ot, nm, hw, fx, kf, ox, px, rx, ui, an, sy, tn, dm, mf, dv, dq] |
| 4 | Crohn's disease.mp. [mp=ti, bt, ab, ot, nm, hw, fx, kf, ox, px, rx, ui, an, sy, tn, dm, mf, dv, dq] |
| 5 | ulcerative colitis.mp. [mp=ti, bt, ab, ot, nm, hw, fx, kf, ox, px, rx, ui, an, sy, tn, dm, mf, dv, dq] |
| 6 | inflammatory bowel disease.mp. [mp=ti, bt, ab, ot, nm, hw, fx, kf, ox, px, rx, ui, an, sy, tn, dm, mf, dv, dq] |
| 7 | IBD.mp. [mp=ti, bt, ab, ot, nm, hw, fx, kf, ox, px, rx, ui, an, sy, tn, dm, mf, dv, dq] |
| 8 | marker*.mp. [mp=ti, bt, ab, ot, nm, hw, fx, kf, ox, px, rx, ui, an, sy, tn, dm, mf, dv, dq] |
| 9 | biomarker*.mp. [mp=ti, bt, ab, ot, nm, hw, fx, kf, ox, px, rx, ui, an, sy, tn, dm, mf, dv, dq] |
| 10 | parameter*.mp. [mp=ti, bt, ab, ot, nm, hw, fx, kf, ox, px, rx, ui, an, sy, tn, dm, mf, dv, dq] |
| 11 | index.mp. [mp=ti, bt, ab, ot, nm, hw, fx, kf, ox, px, rx, ui, an, sy, tn, dm, mf, dv, dq] |
| 12 | 1 or 2 or 3 |
| 13 | 4 or 5 or 6 or 7 |
| 14 | 8 or 9 or 10 or 11 |
| 15 | 12 and 13 and 14 |

The search was restricted to adult human studies, English language, and publication year from 2000 onwards.

**Web of Science**

**("oxidative stress" OR "oxidative damage" OR "antioxida*")** (All Fields) and **(“Crohn’s disease” or “ulcerative colitis” or “inflammatory bowel disease” or “IBD”)** (All Fields) and **(“marker*” or “index” or “parameter*” or “biomarker*”)** (All Fields) and **("men" or "women" or "patient*" or "female*" or "male*" or "subject*" or "adult*")** (All Fields)

We applied the following filters: “English language”, publication year from 2000 onwards, “not review articles”.

Web of Science lacks a direct filter option for "human studies". To narrow down the results to studies conducted on humans, we included specific keywords as recommended in the following link:
<https://f.hubspotusercontent30.net/hubfs/97439/WoS%20human%20studies%20filter.pdf>

**Supplementary table 2*.*** Studies included in the systematic review and meta-analysis assessing oxidative stress-related biomarkers in patients with IBD and healthy controls.

| Author and year | Country | N_a_ [UC/CD] | N_i_ [UC/CD] | N_c_ | IBD subtype | Activity parameter | Evaluated biomarkers |
| --- | --- | --- | --- | --- | --- | --- | --- |
| Achitei 2013 [1] | Romania | 13/8 | 14/6 | 18 | UC/CD | Mayo score/CDAI | GPx, MDA, SOD |
| Akman 2012 [2] | Turkey | 43 | 48 | 45 | IBD | T&W/CDAI | GPx, R-SH |
| Alagozlu 2013 [3] | Turkey | 33 | 26 | 51 | UC | EAI | AOPP, MDA |
| Andoh 2005 [4] | Japan | 12/18 | 22/19 | 20 | UC/CD | CAI/CDAI | Se, SepP |
| Barros 2020 [5] | Brazil | 20 | 27 | 25 | CD | CDAI | GPx1, Se, SepP, TBARS |
| Baskol 2006 [6] [a] | Turkey | 30 | NA | 30 | UC | T&W | PON-1 |
| Baskol 2008 [7] | Turkey | 30 | NA | 30 | UC | T&W | AOPP, MDA, R-SH |
| Beltran 2010 [8] [b] | Spain | 25 | 19+20 | 25 | CD | HBI | CAT, MDA, SOD |
| Boehm 2009 [9] | Poland | 30/37 | 37/15 | 99 | UC/CD | Mayo score/CDAI | PON-1 |
| Boehm 2012 [10] | Poland | 37 | 15 | 99 | CD | CDAI | MDA |
| Bourgonje 2019a [11] [c] | Netherlands | NA | 51 | 27 | CD | HBI | Alb, R-SH, |
| Bourgonje 2019b [12] [d] | Netherlands | 47/31 | NA | 50 | UC/CD | SCCAI/HBI | Alb, R-SH |
| De Silva 2005 [13] | United Kingdom | 7 | NA | 14 | IBD | HBI, SCCAI | Alb |
| D'Odorico 2001 [14] | Italy | 15/10 | 28/23 | 386 | UC/CD | Powell-Tuck index/CDAI | α-Carotene, β-Carotene, β-Cryptoxanthin, Lutein, Lycopene, Total carotenoids, Vit. A, Vit. E, Zeaxanthin |
| Eraldemir 2016 [15] | Turkey | 24 | 25 | 38 | CD | CDAI | MDA, SOD |
| Erichsen 2003 [16] [e] | Norway | 10 | NA | 10 | CD | CDAI | β-Carotene, R-SH, Vit. C, Vit. E |
| Geerling 2000a [17] [f] | Netherlands | NA | 46/23 | 46/23 | UC/CD | T&W /CDAI | Alb, β-Carotene, GPx, Se, Vit. A, Vit. C, Vit. E, Zn |
| Geerling 2000b [18] [g] | Netherlands | NA | 8 | 70 | CD | CDAI | β-Carotene, GPx, Se, Vit. C, Vit. E, Zn |
| Hengstermann 2008 [19] | Germany, Italy, Austria | 35 | 132 | 45 | IBD | CDAI, CAI | Alb, α-Carotene, β-Carotene, β-Cryptoxanthin, Lutein & zeaxanthin, Lycopene, Se, Total carotenoids, Vit. C, Vit. E, Zn |
| Ipek 2022 [20] | Turkey | 30 | 27 | 29 | UC | RI | Alb |
| Kaliora 2007 [21] [h] | Greece | 10 | 10 | 8 | CD | CDAI | TAC |
| Koutroubakis 2004 [22] | Greece | 43/35 | 51/62 | 72 | UC/CD | SCCAI/CDAI | TAC |
| Krzystek-Korpacka 2008 [23] | Poland | 33/38 | 35/12 | 45 | UC/CD | Mayo Disease Activity Index, RI/CDAI | Alb, AOPP, Transferrin |
| Krzystek-Korpacka 2009 [24] | Poland | 27/42 | 42/13 | 81 | UC/CD | CAI/CDAI | Alb, Transferrin |
| Krzystek-Korpacka 2010 [25] | Poland | 42/53 | 51/28 | 105 | UC/CD | CAI, Mayo score/CDAI | CAT, GPx, SOD |
| Kupcova 2012 [26] | Slovakia | 14 | NA | 52 | CD | CDAI | Alb |
| Loveikyte 2023 [27] | Netherlands | 56/66 | NA | 50 | UC/CD | SCCAI, Mayo endoscopic score/ HBI, SES-CD | Alb, R-SH, Transferrin |
| Luceri 2019 [28] | Italy | 54 | NA | 17 | CD | CDAI | AOPP, TAC, TBARS |
| Maor 2008 [29] | Israel | 16 | 27 | 15 | CD | CDAI | β-Carotene, GPx, MDA |
| Matusiewicz 2017 [30] | Poland | 28/36 | 46/27 | 97 | UC/CD | RI/CDAI | Alb, Transferrin |
| Mohammadi 2016 [31] | Iran | 35 | NA | 30 | IBD | RI, HBI | Alb, SOD, Zn |
| Neselioglu 2018 [32] | Turkey | 58 | 20 | 58 | UC | Clinical score, endoscopic core, and laboratory findings | Alb, R-SH |
| Neubauer 2019 [33] | Poland | 30/37 | 41/10 | 57 | UC/CD | CAI/CDAI | Alb, R-SH, SUA, TAC, |
| Owczarek 2010 [34] | Poland | 14/18 | 17/14 | 31/33 | UC/CD | DAI/CDAI | 8-iso-PGF2a |
| Pacal 2010 [35] [i] | Czech Republic | NA | 25 | 88 | CD | CDAI | GPx, MDA, SOD, TAC |
| Pan 2023 [36] [j] | China | 162 | NA | 170 | CD | CDAI, SES-CD | SUA |
| Pinto 2013 [37] [k] | Brazil | NA | 20 | 16 | CD | CDAI | Se |
| Rana 2014 [38] [l] | India | NA | 81 | 85 | UC | UCAI | CAT, SOD |
| Reimund 2000 [39] | France | 26 | NA | 15 | CD | Van Hees activity index | Alb, GPx, Se, SOD, Zn |
| Sampietro 2002 [40] [h] | Italy | 20 | 20 | 134 | CD | CDAI | Vit. A, Vit. E, TAC, TBARS |
| Sen 2016 [41] [h] | Turkey | 30 | 30 | 66 | CD | CDAI | Alb, TBIL |
| Shi 2019 [42] | China | 95 | 5 | 140 | UC | Mayo score | TBIL |
| Su 2019 [43] | China | 53 | 18 | 120 | CD | CDAI | Alb, SUA, TBIL |
| Szczeklik 2016 [44] | Poland | 25 | 22 | 25 | CD | CDAI | GPx, SOD |
| Szczeklik 2018a [45] | Poland | 28 | 19 | 23 | CD | CDAI | PON-1 |
| Szczeklik 2018b [46] | Poland | 30 | 25 | 25 | CD | CDAI | Alb, R-SH, TAC, TBIL |
| Szczeklik 2018c [47] | Poland | 32 | 26 | 26 | CD | CDAI | CAT, R-SH, MDA, TAC |
| Tian 2018 [48] | China | 94 | 76 | 200 | UC | Mayo score | SUA, TBIL |
| Tüzün 2002 [49] | Turkey | 13/6 | 22/6 | 30 | UC/CD | RI/HBI | GPx, MDA |
| Tzivras 2006 [50] [m] | Greece | 31/8 | NA | 19 | UC/CD | CAI, EAI/CDAI | MDA |
| Wendland 2001 [51] | Canada | 13 | 24 | 37 | CD | CDAI | α-Carotene, β-Carotene, β-Cryptoxanthin, F2-isoprostane, GPx, Lutein & zeaxanthin, Lycopene, Se, Vit. A, Vit. C, Vit. E |
| Yuksel 2017 [52] [n] | Turkey | 40/40 | NA | 80 | UC/CD | EAI/CDAI | Alb, PON-1, TAC |
| Zhang 2020 [53] | China | 448 | NA | 308 | UC | T&W | Alb, TBIL |
| Zhu 2019 [54] | China | 80/174 | 21/160 | 51 | UC/CD | Mayo score/CDAI, HBI | SUA |

Abbreviations: 8-iso-PGF2α, 8-iso-prostaglandin F2α; Alb, albumin; AOPP, advanced oxidation protein products; CAI, clinical activity index; CAT, catalase; CD, Crohn’s disease; CDAI, Crohn’s Disease Activity Index; DAI, disease activity index; EAI, endoscopic activity index; GPx, glutathione peroxidase; HBI, Harvey-Bradshaw Index; IBD, inflammatory bowel disease; MDA, malondialdehyde; N_A_, total number of patient with active disease; NA, not applicable; N_C_, total number of healthy controls; N_I_, total number of patients with inactive disease; PON-1, paraoxonase 1; R-SH, free thiols (including cysteine and glutathione); RBC, red blood cells; RI, Rachmilewitz Index; SCCAI, simple clinical colitis activity index; Se, selenium; SepP, selenoprotein P; SES-CD, Simple Endoscopic Score for Crohn’s Disease; SOD, superoxide dismutase; SUA, serum uric acid; TAC, total antioxidant capacity; TBARS, thiobarbituric acid reactive substances; TBIL, total bilirubin; T&W, Truelove and Witts' criteria; UC, ulcerative colitis; UCAI, ulcerative colitis activity index; Vit. A, vitamin A; Vit. C, vitamin C; Vit. E, vitamin E; WBC, white blood cells; Zn, zinc.
[a] – mildly (80%), moderately (13.3%), and severely active disease (6.7%); considered active
[b] – 19 achieved remission (longitudinal); 20 separate inactive group
[c] – »most patients were in clinical remission«; considered inactive
[d] – 76.7% with HBI or SCCAI > 5; considered active
[e] – 80% active; considered active
[f] – 92% inactive UC, 83% inactive CD; considered inactive
[g] – CDAI: 79 (IQR: 47-120); considered inactive
[h] – Longitudinal
[i] – CDAI: 48 [10-154]; considered inactive
[j] – SES-CD > 3 in 152 out of 162 patients, CDAI ≥ 150 in 122 out of 162 patients; considered active
[k] – CDAI: 95.5±15.9; considered inactive
[l] – »Most of the patients were in remission state«; considered inactive
[m] – CDAI: 331.81±117.25, CAI: 1.52 ± 0.26; considered active
[n] – CDAI: 263.8±80.8, EAI: 7±1.9; considered active

[1] D. Achitei, A. Ciobica, G. Balan, E. Gologan, C. Stanciu, G. Stefanescu, Different profile of peripheral antioxidant enzymes and lipid peroxidation in active and non-active inflammatory bowel disease patients., Dig Dis Sci 58 (2013) 1244–9. https://doi.org/10.1007/s10620-012-2510-z.

[2] T. Akman, M. Akarsu, H. Akpinar, H. Resmi, E. Taylan, Erythrocyte deformability and oxidative stress in inflammatory bowel disease., Dig Dis Sci 57 (2012) 458–64. https://doi.org/10.1007/s10620-011-1882-9.

[3] H. Alagozlu, A. Gorgul, A. Bilgihan, C. Tuncer, S. Unal, Increased plasma levels of advanced oxidation protein products (AOPP) as a marker for oxidative stress in patients with active ulcerative colitis., Clin Res Hepatol Gastroenterol 37 (2013) 80–5. https://doi.org/10.1016/j.clinre.2012.03.034.

[4] A. Andoh, K. Hata, O. Inatomi, T. Tsujikawa, M. Sasaki, Y. Fujiyama, M. Hirashima, H. Maeda, K. Takahashi, Serum selenoprotein-P levels in patients with inflammatory bowel disease, Nutrition 21 (2005) 574–579. https://doi.org/10.1016/j.nut.2004.08.025.

[5] S. Barros, M. Dias, M. de Moura, N. Soares, N. Pierote, C. de Araujo, C. Maia, G. Henriques, V. Barros, J. Neto, J. Parente, D. Marreiro, N. Nogueira, Relationship between selenium status and biomarkers of oxidative stress in Crohn’s disease, NUTRITION 74 (2020). https://doi.org/10.1016/j.nut.2020.110762.

[6] G. Baskol, M. Baskol, A. Yurci, O. Ozbakir, M. Yucesoy, Serum paraoxonase 1 activity and malondialdehyde levels in patients with ulcerative colitis., Cell Biochem Funct 24 (2006) 283–6.

[7] M. Baskol, G. Baskol, D. Kocer, O. Ozbakir, M. Yucesoy, Advanced oxidation protein products: a novel marker of oxidative stress in ulcerative colitis., J Clin Gastroenterol 42 (2008) 687–91. https://doi.org/10.1097/MCG.0b013e318074f91f.

[8] B. Beltrán, P. Nos, F. Dasí, M. Iborra, G. Bastida, M. Martínez, J.-E. OʼConnor, G. Sáez, I. Moret, J. Ponce, Mitochondrial dysfunction, persistent oxidative damage, and catalase inhibition in immune cells of naïve and treated Crohnʼs disease:, Inflammatory Bowel Diseases 16 (2010) 76–86. https://doi.org/10.1002/ibd.21027.

[9] D. Boehm, M. Krzystek-Korpacka, K. Neubauer, M. Matusiewicz, I. Berdowska, B. Zielinski, L. Paradowski, A. Gamian, Paraoxonase-1 status in Crohn’s disease and ulcerative colitis., Inflamm Bowel Dis 15 (2009) 93–9. https://doi.org/10.1002/ibd.20582.

[10] D. Boehm, M. Krzystek-Korpacka, K. Neubauer, M. Matusiewicz, L. Paradowski, A. Gamian, Lipid peroxidation markers in Crohn’s disease: the associations and diagnostic value., Clin Chem Lab Med 50 (2012) 1359–66. https://doi.org/10.1515/cclm-2011-0817.

[11] A. Bourgonje, J. von Martels, M. Bulthuis, M. van Londen, K. Faber, G. Dijkstra, H. van Goor, Crohn’s Disease in Clinical Remission Is Marked by Systemic Oxidative Stress, FRONTIERS IN PHYSIOLOGY 10 (2019). https://doi.org/10.3389/fphys.2019.00499.

[12] A. Bourgonje, R. Gabriels, M. de Borst, M. Bulthuis, K. Faber, H. van Goor, G. Dijkstra, Serum Free Thiols Are Superior to Fecal Calprotectin in Reflecting Endoscopic Disease Activity in Inflammatory Bowel Disease, ANTIOXIDANTS 8 (2019). https://doi.org/10.3390/antiox8090351.

[13] A. De Silva, E. Tsironi, R. Feakins, D. Rampton, Efficacy and tolerability of oral iron therapy in inflammatory bowel disease: a prospective, comparative trial, ALIMENTARY PHARMACOLOGY & THERAPEUTICS 22 (2005) 1097–1105. https://doi.org/10.1111/j.1365-2036.2005.02700.x.

[14] A. D’Odorico, S. Bortolan, R. Cardin, R. D’Inca’, D. Martines, A. Ferronato, G.C. Sturniolo, Reduced plasma antioxidant concentrations and increased oxidative DNA damage in inflammatory bowel disease., Scand J Gastroenterol 36 (2001) 1289–94.

[15] F. Eraldemir, M. Musul, A. Duman, B. Oztas, C. Baydemir, S. Hulagu, The relationship between neutrophil/lymphocyte and platelet/lymphocyte ratios with oxidative stress in active Crohn’s disease patients, HIPPOKRATIA 20 (2016) 268–273.

[16] K. Erichsen, T. Hausken, R. Ulvik, A. Svardal, A. Berstad, R. Berge, Ferrous fumarate deteriorated plasma antioxidant status in patients with Crohn disease, SCANDINAVIAN JOURNAL OF GASTROENTEROLOGY 38 (2003) 543–548. https://doi.org/10.1080/00365520310000771.

[17] B.J. Geerling, A. Badart-Smook, R.W. Stockbrugger, R.-J.M. Brummer, Comprehensive nutritional status in recently diagnosed patients with inflammatory bowel disease compared with population controls, Eur. J. Clin. Nutr. 54 (2000) 514–521. https://doi.org/10.1038/sj.ejcn.1601049.

[18] B.J. Geerling, A. Badart-Smook, C. van Deursen, A.C. van Houwelingen, M.G. Russel, R.W. Stockbrugger, R.J. Brummer, Nutritional supplementation with N-3 fatty acids and antioxidants in patients with Crohn’s disease in remission: effects on antioxidant status and fatty acid profile., Inflamm Bowel Dis 6 (2000) 77–84.

[19] S. Hengstermann, L. Valentini, L. Schaper, C. Buning, T. Koernicke, M. Maritschnegg, S. Buhner, W. Tillinger, N. Regano, F. Guglielmi, B. Winklhofer-Roob, H. Lochs, Altered status of antioxidant vitamins and fatty acids in patients with inactive inflammatory bowel disease, CLINICAL NUTRITION 27 (2008) 571–578. https://doi.org/10.1016/j.clnu.2008.01.007.

[20] Ipek S., Yalcin H., Toprak B., Association between disease activity and ischaemia-modified albumin in patients with ulcerative colitis, Prz. Gastroenterol. 17 (2022) 203–206. https://doi.org/10.5114/pg.2021.109664.

[21] A.C. Kaliora, M.G. Stathopoulou, J.K. Triantafillidis, G.V.Z. Dedoussis, N.K. Andrikopoulos, Chios mastic treatment of patients with active Crohn’s disease., World J Gastroenterol 13 (2007) 748–53.

[22] I. Koutroubakis, N. Malliaraki, P. Dimoulios, K. Karmiris, E. Castanas, E. Kouroumalis, Decreased total and corrected antioxidant capacity in patients with inflammatory bowel disease, DIGESTIVE DISEASES AND SCIENCES 49 (2004) 1433–1437. https://doi.org/10.1023/B:DDAS.0000042242.22898.d9.

[23] M. Krzystek-Korpacka, K. Neubauer, I. Berdowska, D. Boehm, B. Zielinski, P. Petryszyn, G. Terlecki, L. Paradowski, A. Gamian, Enhanced formation of advanced oxidation protein products in IBD, INFLAMMATORY BOWEL DISEASES 14 (2008) 794–802. https://doi.org/10.1002/ibd.20383.

[24] M. Krzystek-Korpacka, K. Neubauer, M. Matusiewicz, Platelet-derived growth factor-BB reflects clinical, inflammatory and angiogenic disease activity and oxidative stress in inflammatory bowel disease., Clin Biochem 42 (2009) 1602–9. https://doi.org/10.1016/j.clinbiochem.2009.08.002.

[25] M. Krzystek-Korpacka, K. Neubauer, I. Berdowska, B. Zielinski, L. Paradowski, A. Gamian, Impaired erythrocyte antioxidant defense in active inflammatory bowel disease: impact of anemia and treatment., Inflamm Bowel Dis 16 (2010) 1467–75. https://doi.org/10.1002/ibd.21234.

[26] V. Kupcova, L. Turecky, E. Uhlikova, The role of oxidative stress in anti-tumor necrosis factor antibody treatment in Crohn’s disease., Curr Med Chem 19 (2012) 5226–31.

[27] R. Loveikyte, A. Bourgonje, J. van der Reijden, M. Bulthuis, L. Hawinkels, M. Visschedijk, E. Festen, H. van Dullemen, R. Weersma, H. van Goor, A. Jong, G. Dijkstra, Hepcidin and Iron Status in Patients With Inflammatory Bowel Disease Undergoing Induction Therapy With Vedolizumab or Infliximab, INFLAMMATORY BOWEL DISEASES 29 (2023) 1272–1284. https://doi.org/10.1093/ibd/izad010.

[28] C. Luceri, E. Bigagli, S. Agostiniani, F. Giudici, D. Zambonin, S. Scaringi, F. Ficari, M. Lodovici, C. Malentacchi, Analysis of Oxidative Stress-Related Markers in Crohn’s Disease Patients at Surgery and Correlations with Clinical Findings, Antioxidants 8 (2019) 378. https://doi.org/10.3390/antiox8090378.

[29] I. Maor, T. Rainis, A. Lanir, A. Lavy, Oxidative stress, inflammation and neutrophil superoxide release in patients with Crohn’s disease: distinction between active and non-active disease., Dig Dis Sci 53 (2008) 2208–14. https://doi.org/10.1007/s10620-007-0141-6.

[30] M. Matusiewicz, K. Neubauer, P. Lewandowska, A. Gamian, M. Krzystek-Korpacka, Reduced Transferrin Levels in Active Inflammatory Bowel Disease., Biomed Res Int 2017 (2017) 9541370. https://doi.org/10.1155/2017/9541370.

[31] E. Mohammadi, D. Qujeq, H. Taheri, K. Hajian-Tilaki, Evaluation of Serum Trace Element Levels and Superoxide Dismutase Activity in Patients with Inflammatory Bowel Disease: Translating Basic Research into Clinical Application, Biol Trace Elem Res 177 (2017) 235–240. https://doi.org/10.1007/s12011-016-0891-0.

[32] S. Neselioglu, P.B. Keske, A.A. Senat, O.T. Yurekli, S. Erdogan, M. Alisik, M. Ergin, H. Koseoglu, O. Ersoy, O. Erel, The relationship between severity of ulcerative colitis and thiol-disulphide homeostasis, BRATISLAVA MEDICAL JOURNAL-BRATISLAVSKE LEKARSKE LISTY 119 (2018) 498–502. https://doi.org/10.4149/BLL_2018_091.

[33] K. Neubauer, R. Kempinski, M. Matusiewicz, I. Bednarz-Misa, M. Krzystek-Korpacka, Nonenzymatic Serum Antioxidant Capacity in IBD and Its Association with the Severity of Bowel Inflammation and Corticosteroids Treatment., Medicina (Kaunas) 55 (2019). https://doi.org/10.3390/medicina55040088.

[34] D. Owczarek, D. Cibor, T. Mach, Asymmetric dimethylarginine (ADMA), symmetric dimethylarginine (SDMA), arginine, and 8-iso-prostaglandin F2alpha (8-iso-PGF2alpha) level in patients with inflammatory bowel diseases., Inflamm Bowel Dis 16 (2010) 52–7. https://doi.org/10.1002/ibd.20994.

[35] L. Pacal, K. Kankova, J. Varvarovska, J. Sykora, J. Kozeluhova, Z. Rusavy, J. Racek, R. Stetina, Crohn’s disease activity versus extent of DNA damage/repair and variability in the rage gene, Scr. Med. Fac. Med. Univ. Brun. Masarykianae 83 (2010) 72–80.

[36] Y. Pan, X. Huang, Z. Zhou, X. Yang, L. Li, C. Gao, Y. Zhang, Y. Zhang, Clinical significance of a novel uric-acid-based biomarker in the prediction of disease activity and response to infliximab therapy in Crohn’s disease, Scandinavian Journal of Gastroenterology 58 (2023) 737–743. https://doi.org/10.1080/00365521.2023.2175181.

[37] M.A.S. Pinto, M.S.-M.S. Lopes, S.T.O. Bastos, C.L.L. Reigada, R.F. Dantas, J.C.B. Neto, A.S. Luna, K. Madi, T. Nunes, C. Zaltman, Does active Crohn’s disease have decreased intestinal antioxidant capacity?., J Crohns Colitis 7 (2013) e358-66. https://doi.org/10.1016/j.crohns.2013.02.010.

[38] S. Rana, S. Sharma, K. Prasad, S. Sinha, K. Singh, Role of oxidative stress & antioxidant defence in ulcerative colitis patients from north India, INDIAN JOURNAL OF MEDICAL RESEARCH 139 (2014) 568–571.

[39] J. Reimund, C. Hirth, C. Koehl, R. Baumann, B. Duclos, Antioxidant and immune status in active Crohn’s disease. A possible relationship, CLINICAL NUTRITION 19 (2000) 43–48. https://doi.org/10.1054/clnu.1999.0073.

[40] G. Sampietro, M. Cristaidi, G. Cervato, G. Maconi, P. Danelli, R. Cervellione, M. Rovati, G. Porro, B. Cestaro, A. Taschieri, Oxidative stress, vitamin A and vitamin E behaviour in patients submitted to conservative surgery for complicated Crohn’s disease, DIGESTIVE AND LIVER DISEASE 34 (2002) 696–701. https://doi.org/10.1016/S1590-8658(02)80020-2.

[41] H. Sen, E. Akbal, G. Erbag, E. Binnetoglu, Relationship Between Bilirubin Level and Disease Activity in Crohn’s Disease, JOURNAL OF CLINICAL AND ANALYTICAL MEDICINE 7 (2016) 27–30. https://doi.org/10.4328/JCAM.4506.

[42] H. Shi, Y. Feng, J. Jiang, J. Zhao, X. Li, X. Liu, Correlations between the serum bilirubin level and ulcerative colitis: a case-control study., Eur J Gastroenterol Hepatol 31 (2019) 992–997. https://doi.org/10.1097/MEG.0000000000001466.

[43] Q. Su, X. Li, W. Mo, Z. Yang, Low serum bilirubin, albumin, and uric acid levels in patients with Crohn’s disease, MEDICINE 98 (2019). https://doi.org/10.1097/MD.0000000000015664.

[44] K. Szczeklik, W. Krzysciak, R. Domagala-Rodacka, P. Mach, D. Darczuk, D. Cibor, J. Pytko-Polonczyk, T. Rodacki, D. Owczarek, ALTERATIONS IN GLUTATHIONE PEROXIDASE AND SUPEROXIDE DISMUTASE ACTIVITIES IN PLASMA AND SALIVA IN RELATION TO DISEASE ACTIVITY IN PATIENTS WITH CROHN’S DISEASE, JOURNAL OF PHYSIOLOGY AND PHARMACOLOGY 67 (2016) 709–715.

[45] K. Szczeklik, T. Mach, D. Cibor, D. Owczarek, J. Sapa, M. Papiez, J. Pytko-Polonczyk, W. Krzysciak, Correlation of Paraoxonase-1 with the Severity of Crohn’s Disease, MOLECULES 23 (2018). https://doi.org/10.3390/molecules23102603.

[46] K. Szczeklik, W. Krzysciak, D. Cibor, K. Koziol, H. Pocztar, J. Pytko-Polonczyk, T. Mach, D. Owczarek, Evaluation of plasma concentrations of selected antioxidant parameters in patients with active Crohn’s disease., Folia Med Cracov 58 (2018) 119–130.

[47] K. Szczeklik, W. Krzysciak, D. Cibor, R. Domagala-Rodacka, J. Pytko-Polonczyk, T. Mach, D. Owczarek, Markers of lipid peroxidation and antioxidant status in the serum and saliva of patients with active Crohn disease., Pol Arch Intern Med 128 (2018) 362–370. https://doi.org/10.20452/pamw.4273.

[48] S. Tian, J. Li, R. Li, Z. Liu, W. Dong, Decreased Serum Bilirubin Levels and Increased Uric Acid Levels are Associated with Ulcerative Colitis, MEDICAL SCIENCE MONITOR 24 (2018) 6298–6304. https://doi.org/10.12659/MSM.909692.

[49] A. Tüzün, A. Erdil, V. İnal, A. Aydın, S. Bağcı, Z. Yeşilova, A. Sayal, N. Karaeren, K. Dağalp, Oxidative stress and antioxidant capacity in patients with inflammatory bowel disease, Clinical Biochemistry 35 (2002) 569–572. https://doi.org/10.1016/S0009-9120(02)00361-2.

[50] M. Tzivras, V. Koussoulas, E.J. Giamarellos-Bourboulis, D. Tzivras, T. Tsaganos, P. Koutoukas, H. Giamarellou, A. Archimandritis, Role of soluble triggering receptor expressed on myeloid cells in inflammatory bowel disease., World J Gastroenterol 12 (2006) 3416–9.

[51] B. Wendland, E. Aghdassi, C. Tam, J. Carrrier, A. Steinhart, S. Wolman, D. Baron, J. Allard, Lipid peroxidation and plasma antioxidant micronutrients in Crohn disease, AMERICAN JOURNAL OF CLINICAL NUTRITION 74 (2001) 259–264.

[52] M. Yuksel, M. Kaplan, Y.O. Ozin, Z.M.Y. Kilic, E. Kayacetin, I. Ates, M.F. Arikan, C. Topcuoglu, Is Oxidative Stress Associated with Activation and Pathogenesis of Inflammatory Bowel Disease?, J. Med. Biochem. 36 (2017) 341–348. https://doi.org/10.1515/jomb-2017-0013.

[53] M. Zhang, H. Wang, Y. Shi, J. Zhou, W. Yan, T. Ma, S. Wu, X. Yang, Efficacy of Serum Total Bilirubin in Predicting the Severity of Ulcerative Colitis: A Cross-Sectional Study, ANNALS OF CLINICAL AND LABORATORY SCIENCE 50 (2020) 228–232.

[54] F. Zhu, D. Feng, T. Zhang, L. Gu, W. Zhu, Z. Guo, Y. Li, N. Lu, J. Gong, N. Li, Altered uric acid metabolism in isolated colonic Crohn’s disease but not ulcerative colitis, J of Gastro and Hepatol 34 (2019) 154–161. https://doi.org/10.1111/jgh.14356.

**Supplementary figure 1.** Forest plots of oxidative stress-related biomarkers in patients with active and inactive IBD versus healthy controls.

1. 8-iso-PGF2α


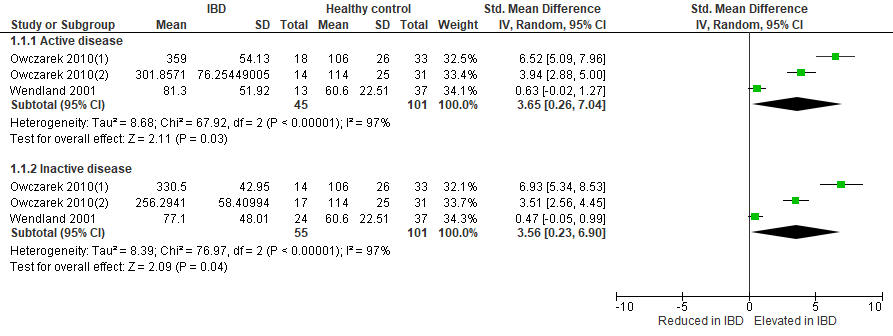
 (1) cohort of patients with Crohn’s disease; (2) cohort of patients with ulcerative colitis

1. AOPP


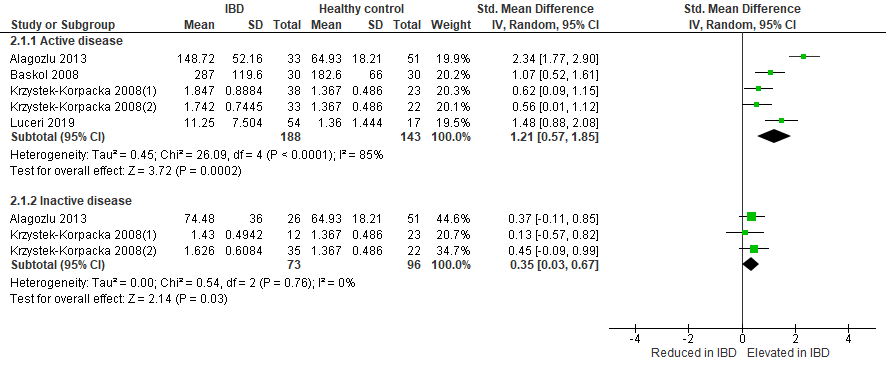


(1) cohort of patients with Crohn’s disease; (2) cohort of patients with ulcerative colitis

1. MDA


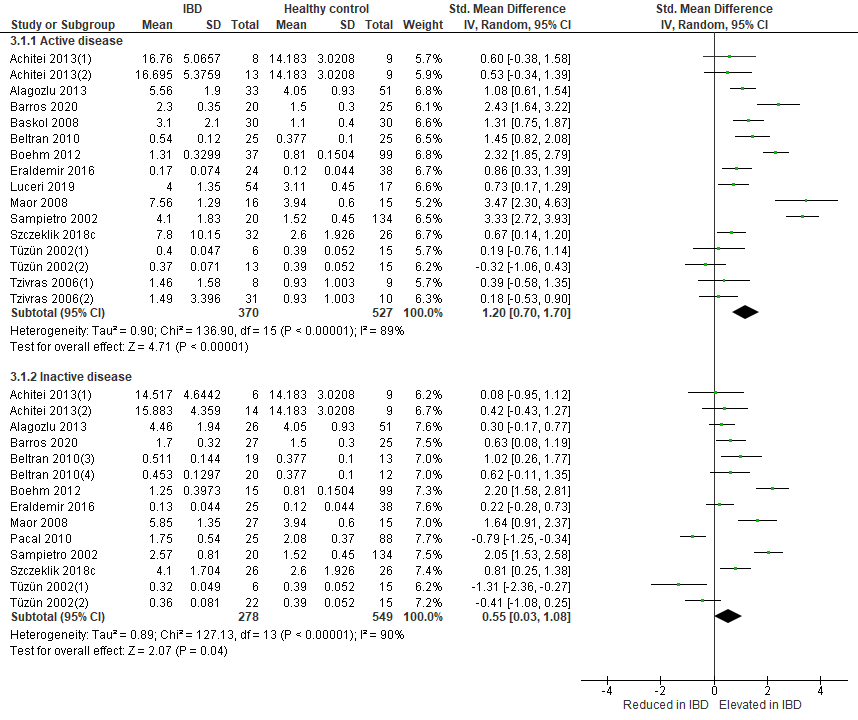
(1) cohort of patients with Crohn’s disease (2) ulcerative colitis patient cohort; (3) longitudinal cohort of patients with inactive disease; (4) cross-sectional cohort of patients with inactive disease

1. PON-1


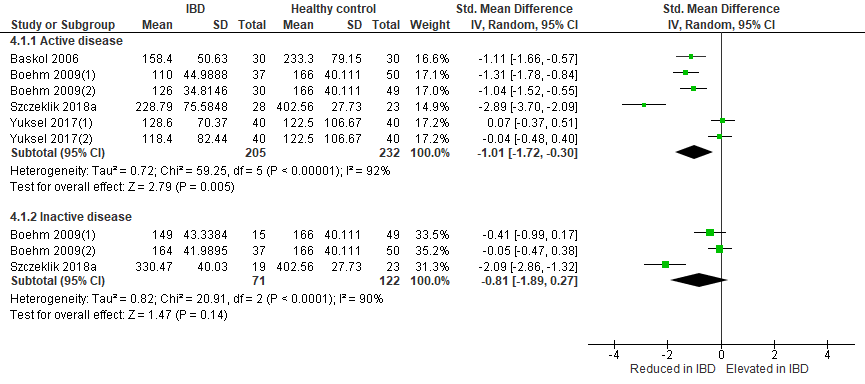
(1) cohort of patients with Crohn’s disease; (2) cohort of patients with ulcerative colitis

1. GPx (plasma or serum)


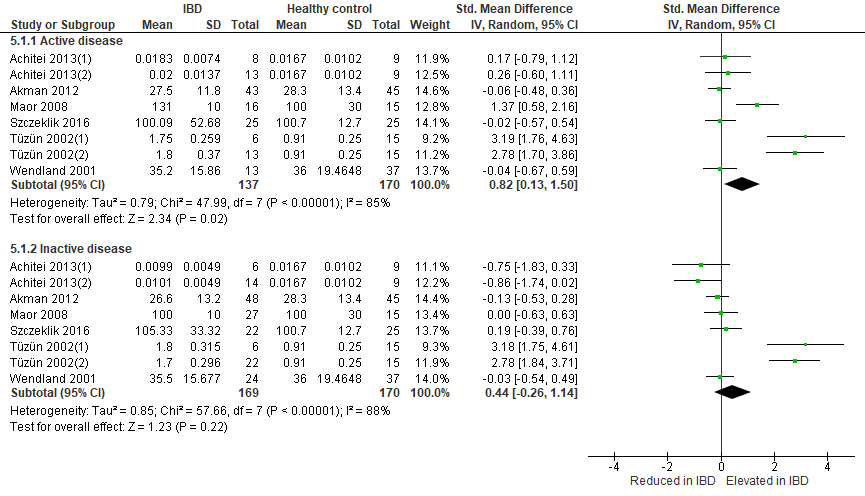
(1) cohort of patients with Crohn’s disease; (2) ulcerative colitis patient cohort

1. GPx (RBC)


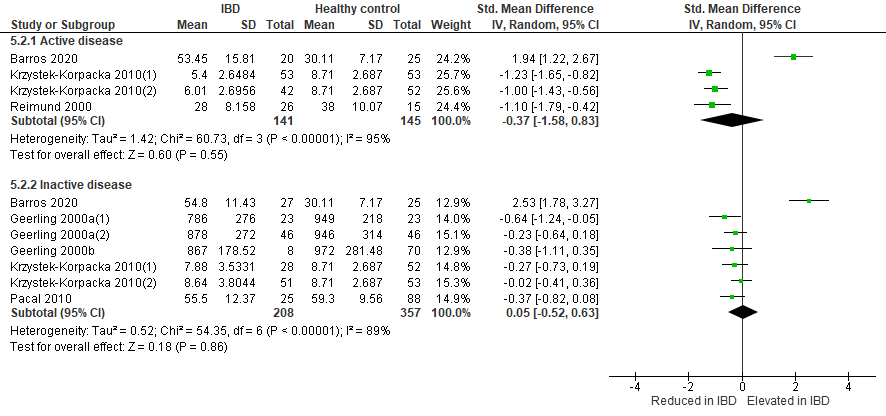
(1) cohort of patients with Crohn’s disease; (2) cohort of patients with ulcerative colitis

1. CAT


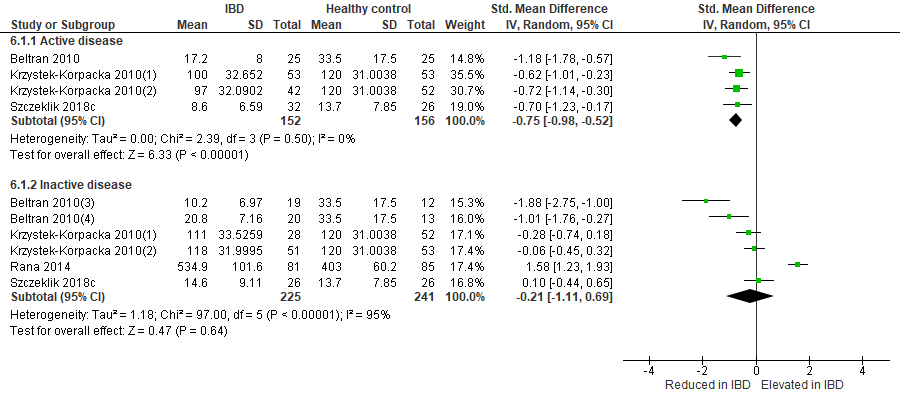
(1) cohort of patients with Crohn’s disease; (2) cohort of patients with ulcerative colitis; (3) longitudinal cohort of patients with inactive disease; (4) cross-sectional cohort of patients with inactive disease

1. SOD (plasma or serum)


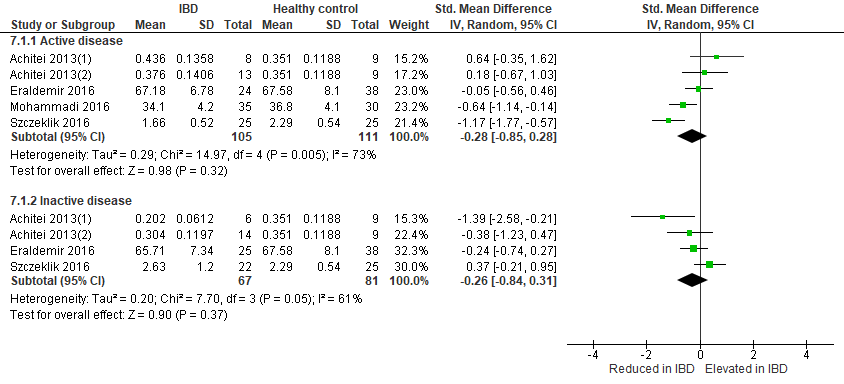
(1) cohort of patients with Crohn’s disease; (2) cohort of patients with ulcerative colitis

1. SOD (RBC or WBC)


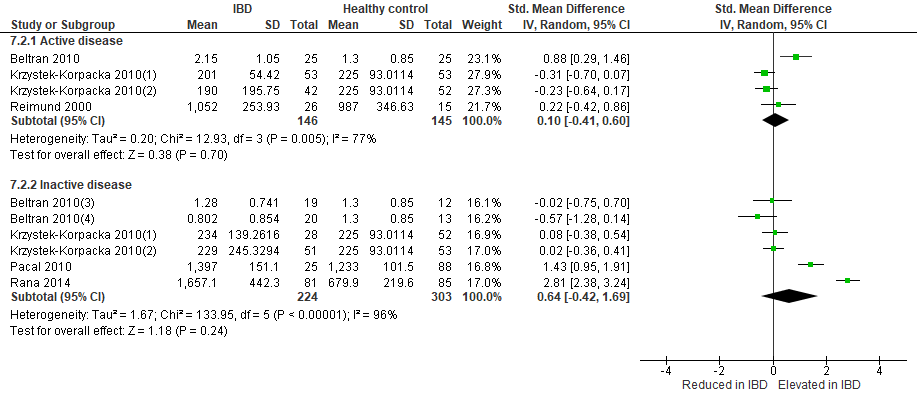
(1) cohort of patients with Crohn’s disease; (2) cohort of patients with ulcerative colitis; (3) longitudinal cohort of patients with inactive disease; (4) cross-sectional cohort of patients with inactive disease

1. β-Carotene


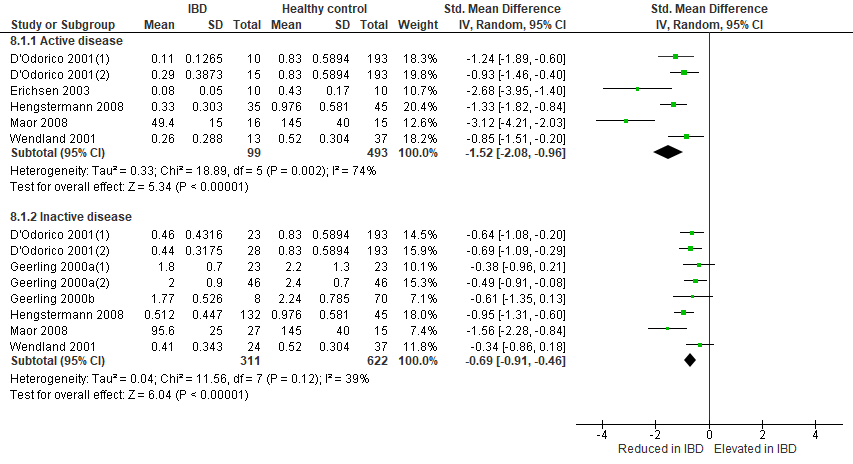
(1) cohort of patients with Crohn’s disease; (2) cohort of patients with ulcerative colitis

1. Lycopene


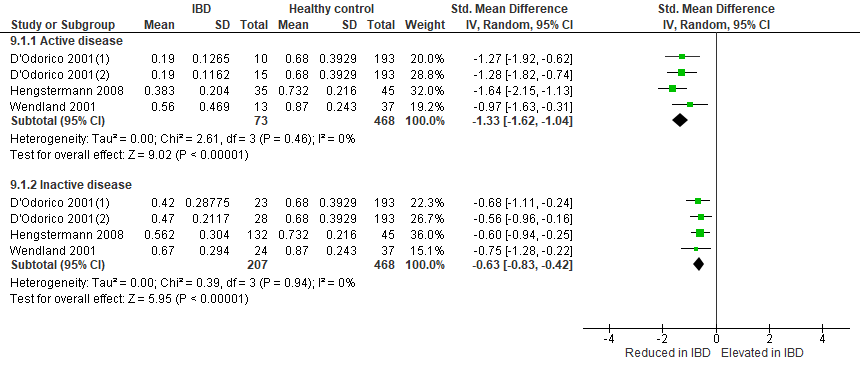
(1) cohort of patients with Crohn’s disease; (2) cohort of patients with ulcerative colitis

1. Total carotenoids


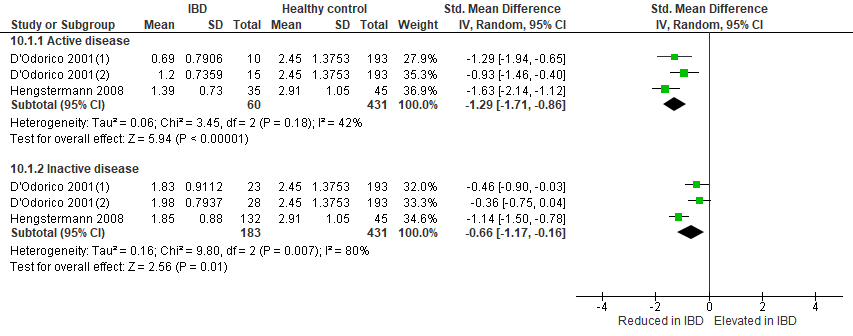
(1) cohort of patients with Crohn’s disease; (2) cohort of patients with ulcerative colitis

1. Vitamin A


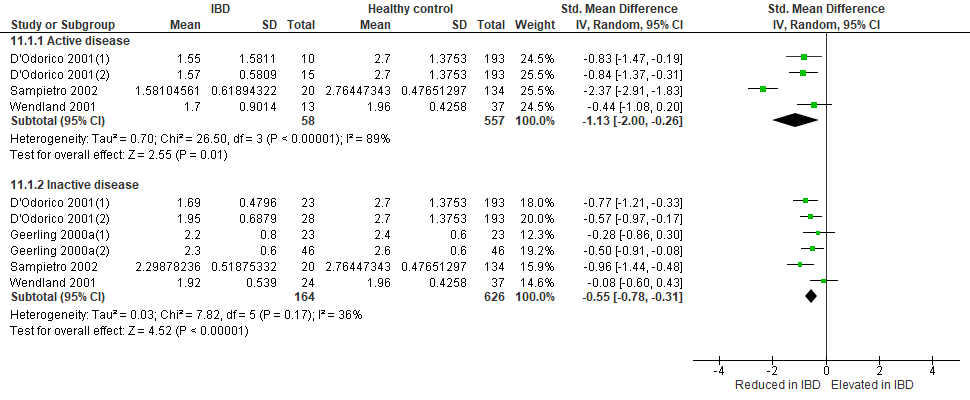
(1) cohort of patients with Crohn’s disease; (2) cohort of patients with ulcerative colitis

1. Vitamin C


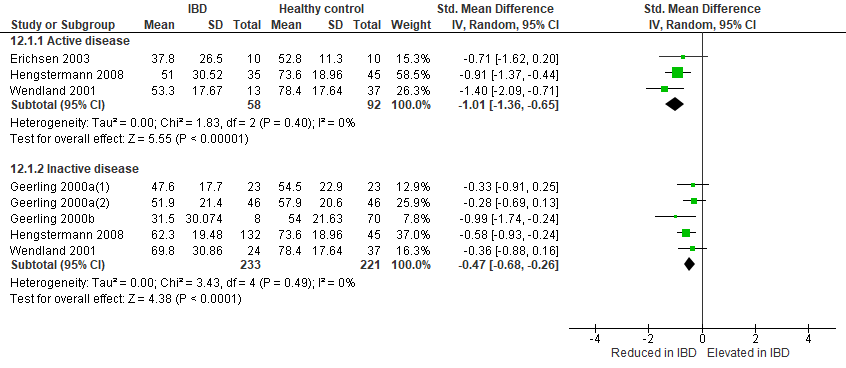
(1) cohort of patients with Crohn’s disease; (2) cohort of patients with ulcerative colitis

1. β-Cryptoxanthin


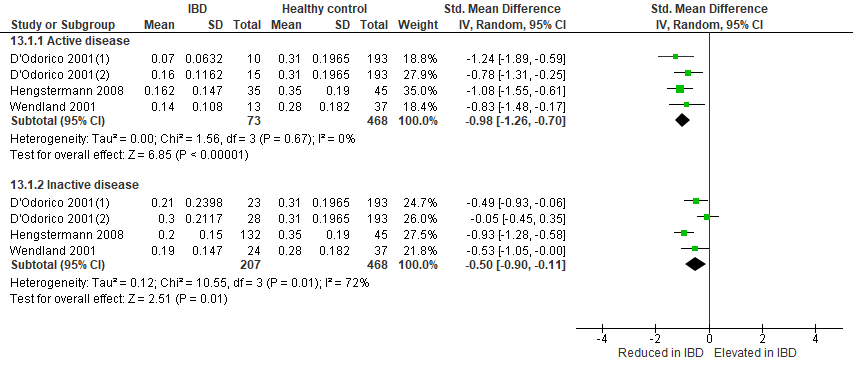
(1) Cohort of patients with Crohn’s disease; (2) cohort of patients with ulcerative colitis

1. Lutein and zeaxanthin


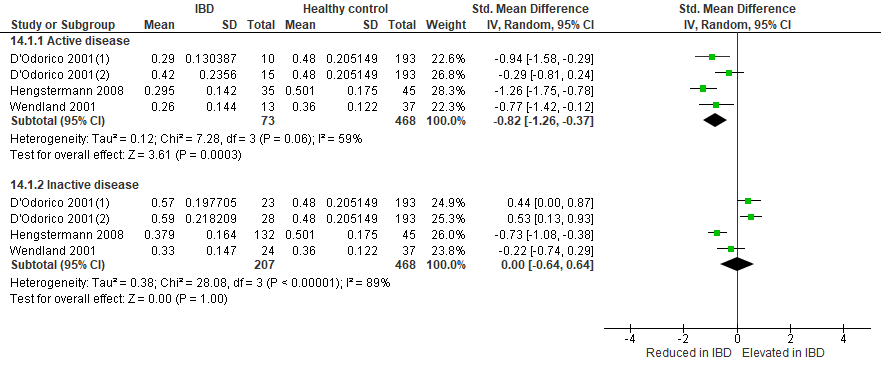
(1) cohort of patients with Crohn’s disease; (2) cohort of patients with ulcerative colitis

1. α-Carotene


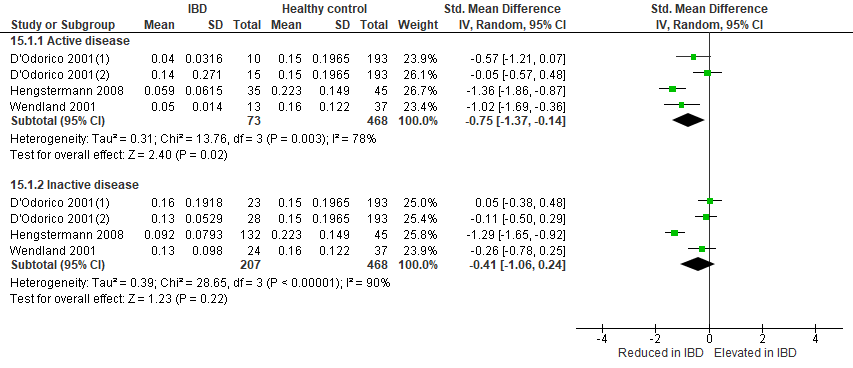
(1) cohort of patients with Crohn’s disease; (2) cohort of patients with ulcerative colitis

1. Vitamin E


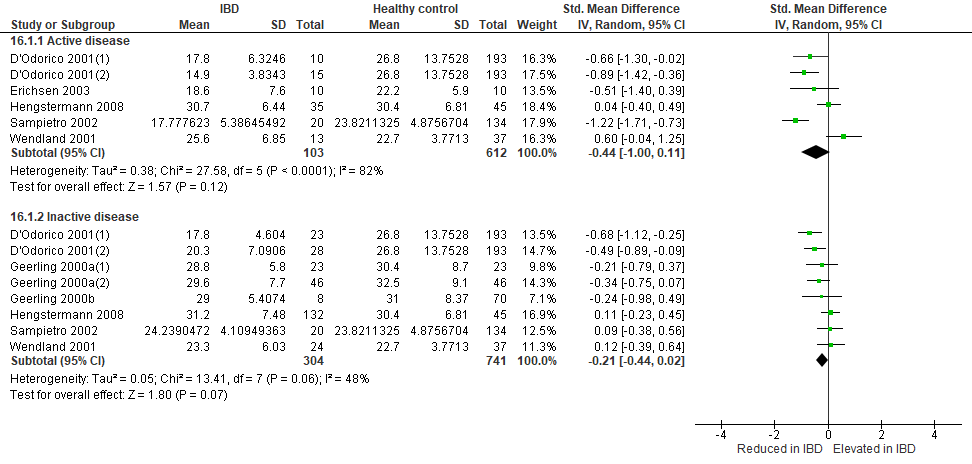
(1) cohort of patients with Crohn’s disease; (2) cohort of patients with ulcerative colitis

1. Albumin


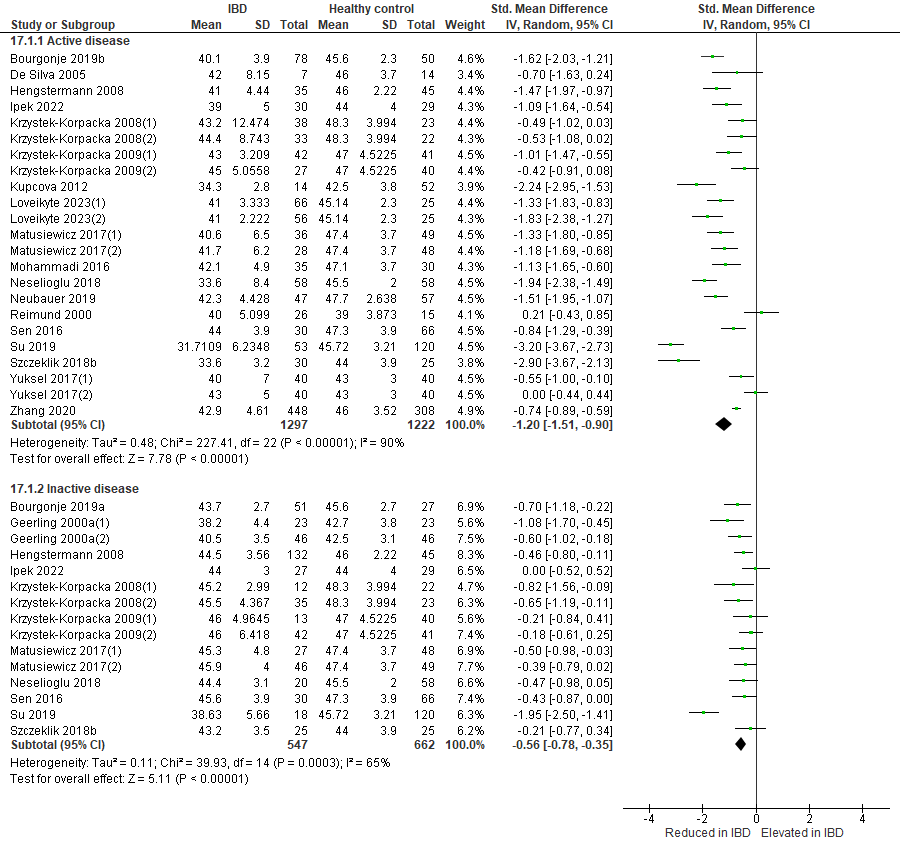
 (1) cohort of patients with Crohn’s disease; (2) cohort of patients with ulcerative colitis

1. SepP


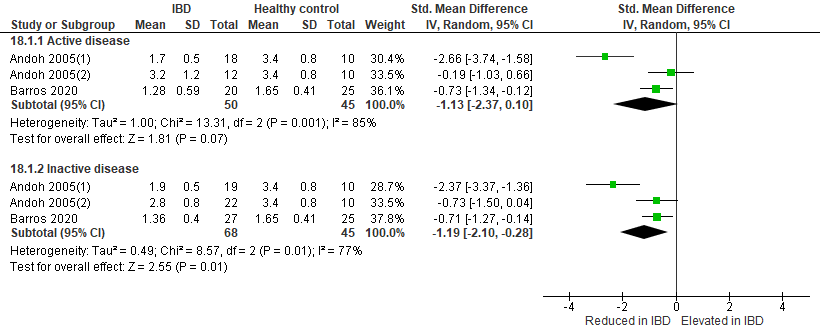
(1) cohort of patients with Crohn’s disease; (2) cohort of patients with ulcerative colitis

1. Transferrin


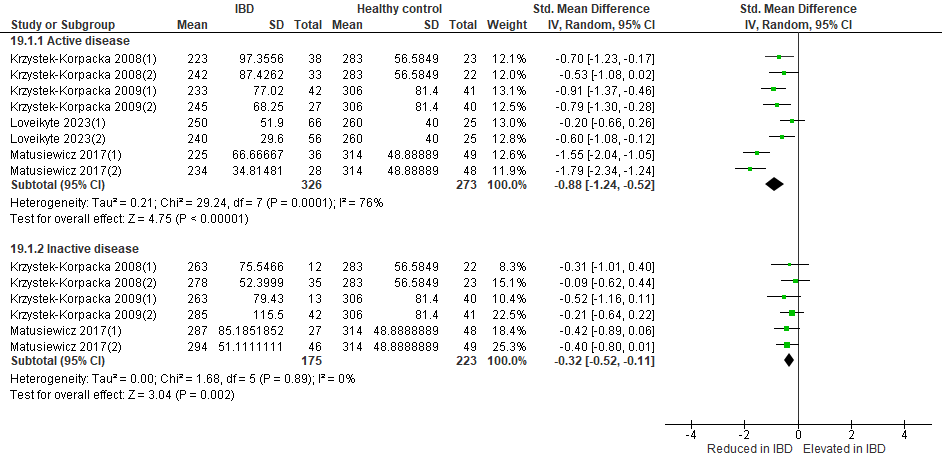


(1) cohort of patients with Crohn’s disease; (2) cohort of patients with ulcerative colitis

1. R-SH


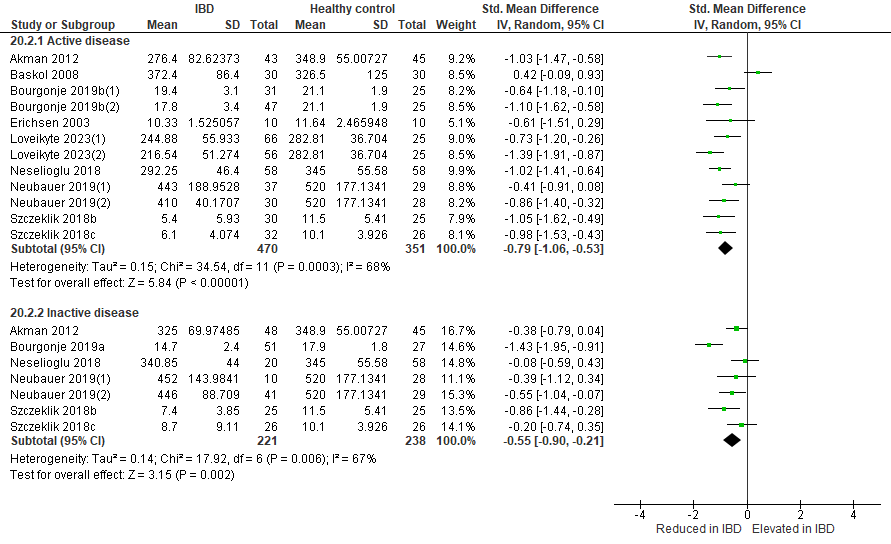
 (1) cohort of patients with Crohn’s disease; (2) cohort of patients with ulcerative colitis

1. TAC


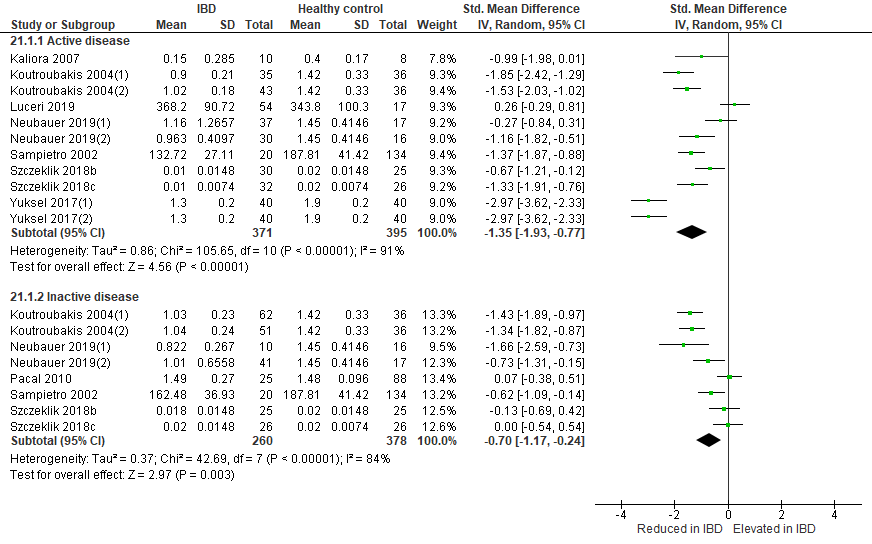
(1) cohort of patients with Crohn’s disease; (2) cohort of patients with ulcerative colitis

1. TBIL


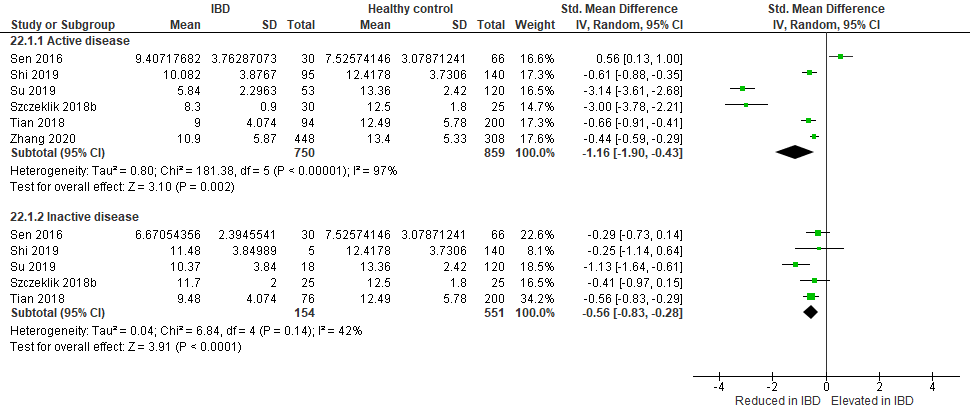


1. Se


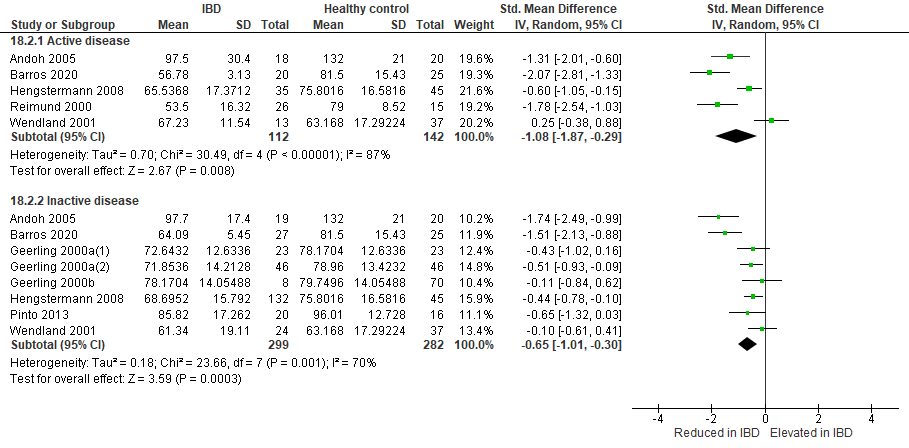
(1) cohort of patients with Crohn’s disease; (2) cohort of patients with ulcerative colitis

1. Zn


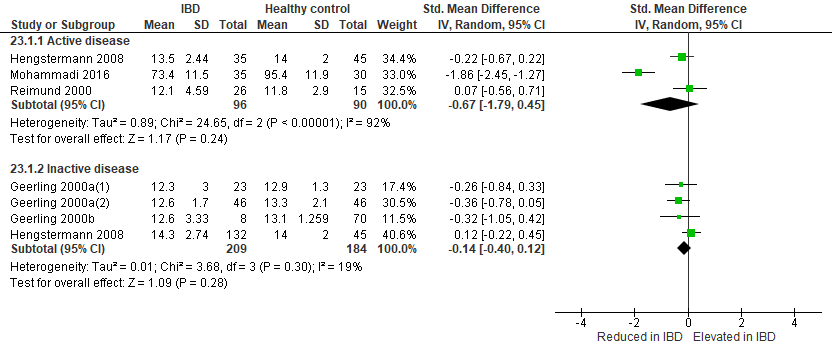


(1) cohort of patients with Crohn’s disease; (2) cohort of patients with ulcerative colitis

1. SUA


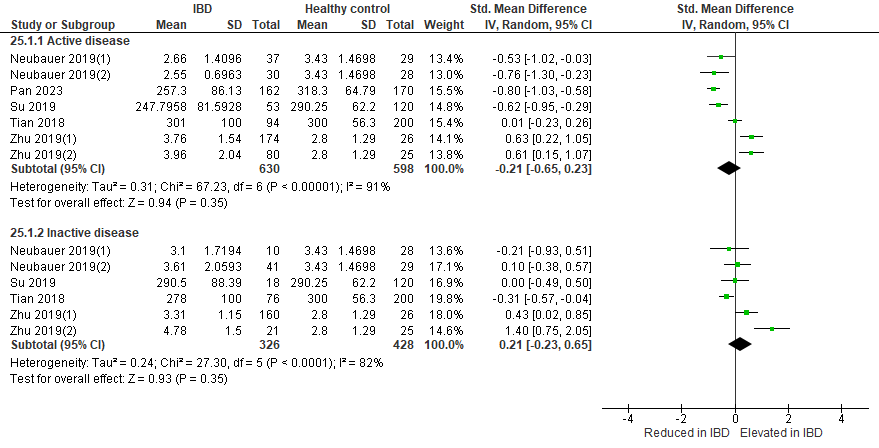


(1) cohort of patients with Crohn’s disease; (2) cohort of patients with ulcerative colitis

**Supplementary figure 2.** Forest plots comparing oxidative stress-related biomarkers in patients with active CD and UC versus those with inactive disease states.

1. 8-iso-PGF2α


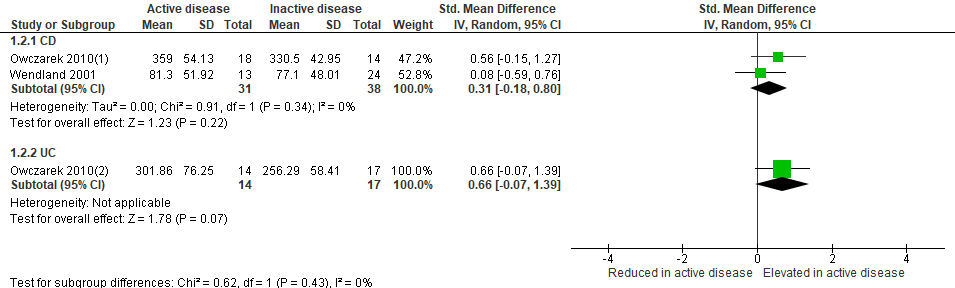
(1) cohort of patients with Crohn’s disease; (2) cohort of patients with ulcerative colitis

1. AOPP


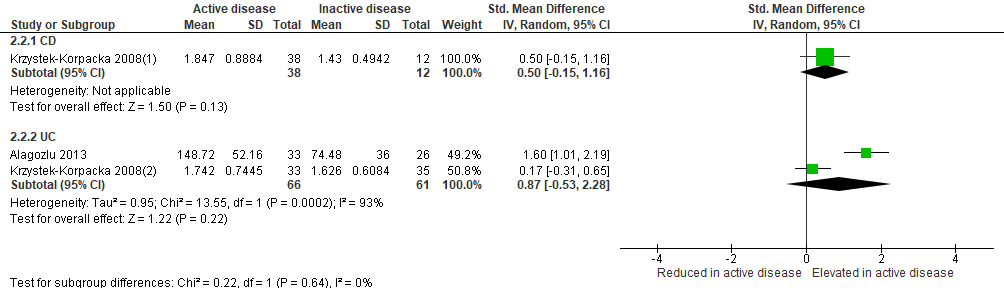
(1) cohort of patients with Crohn’s disease; (2) cohort of patients with ulcerative colitis

1. MDA


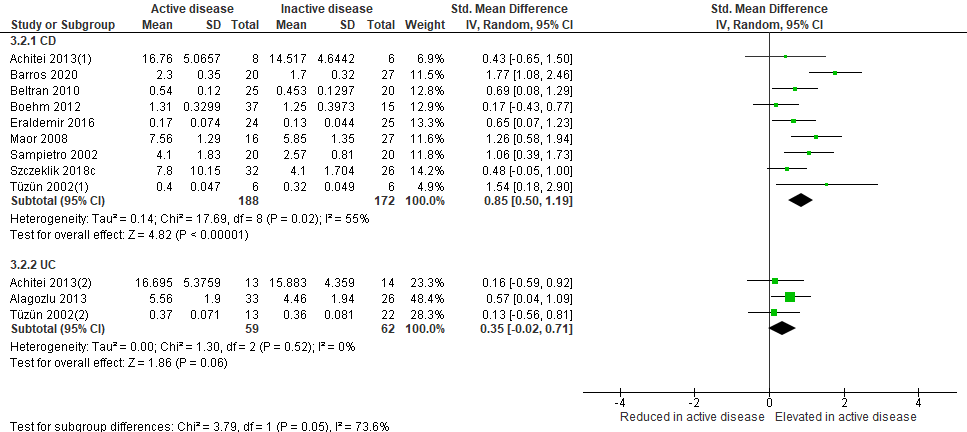
(1) cohort of patients with Crohn’s disease; (2) cohort of patients with ulcerative colitis

1. PON-1


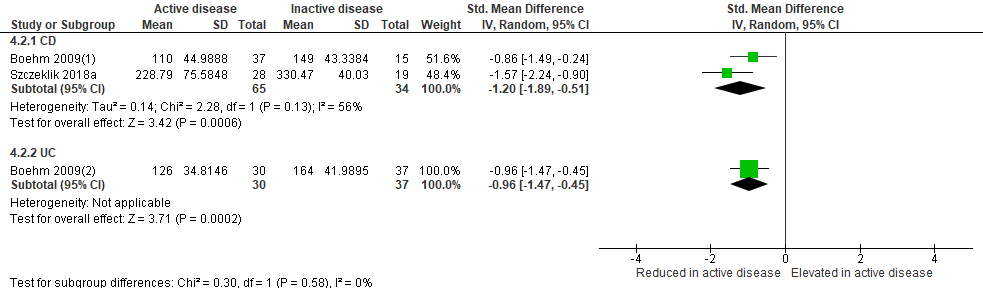
(1) cohort of patients with Crohn’s disease; (2) cohort of patients with ulcerative colitis

1. GPx (plasma or serum)


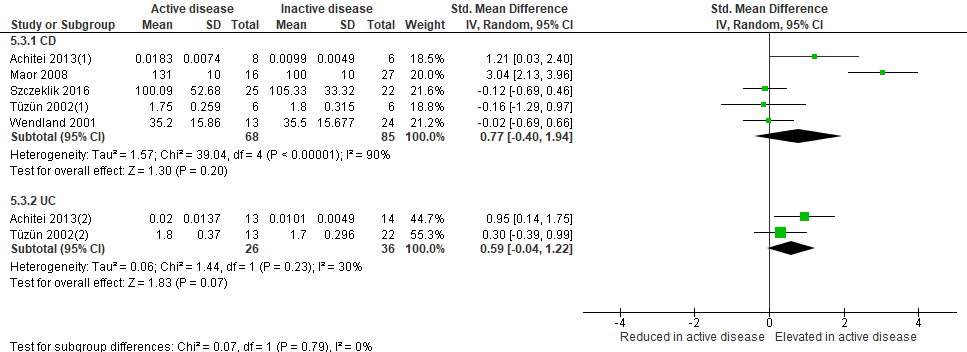
(1) cohort of patients with Crohn’s disease; (2) cohort of patients with ulcerative colitis

1. GPx (RBC)


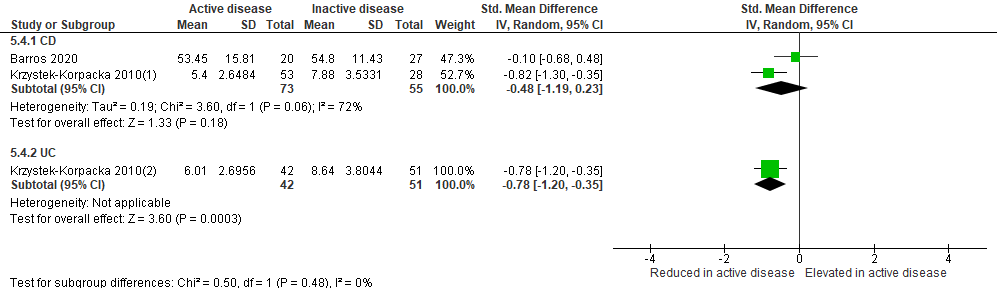
(1) cohort of patients with Crohn’s disease; (2) cohort of patients with ulcerative colitis

1. CAT


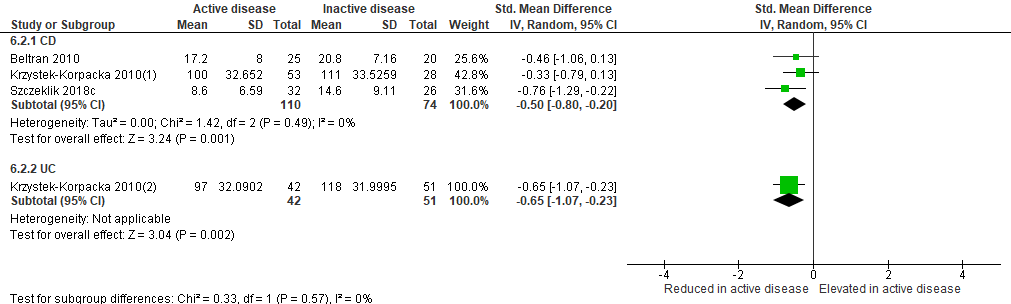
(1) cohort of patients with Crohn’s disease; (2) cohort of patients with ulcerative colitis

1. SOD (plasma or serum)


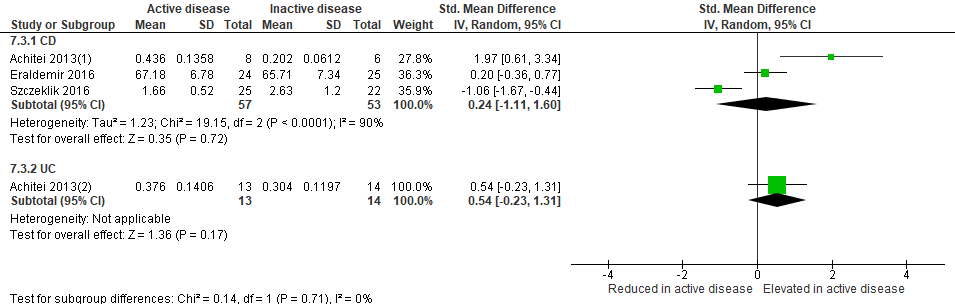
(1) cohort of patients with Crohn’s disease; (2) cohort of patients with ulcerative colitis

1. SOD (RBC or WBC)


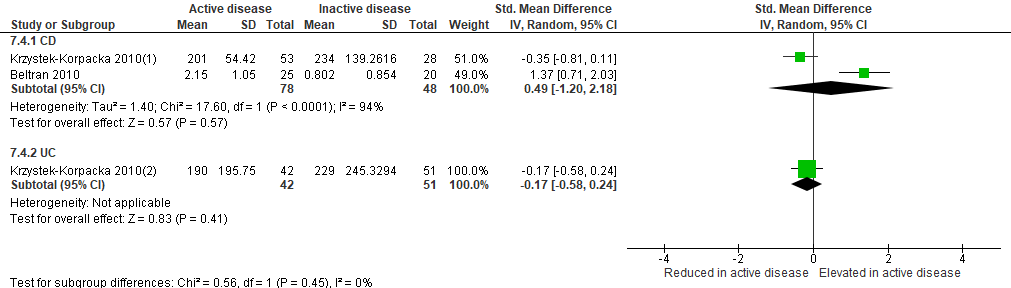
(1) cohort of patients with Crohn’s disease; (2) cohort of patients with ulcerative colitis

1. β-Carotene


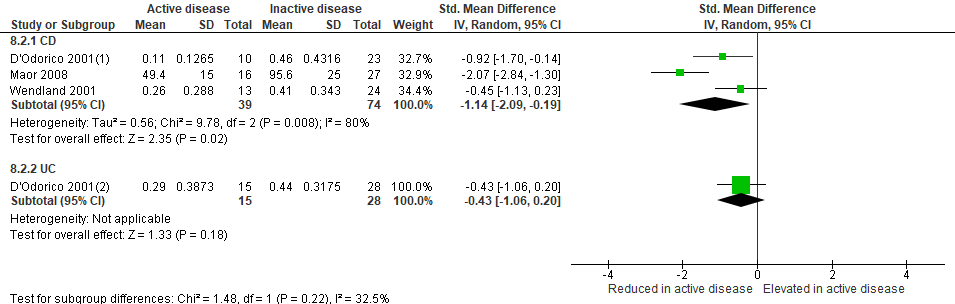
(1) cohort of patients with Crohn’s disease; (2) cohort of patients with ulcerative colitis

1. Lycopene


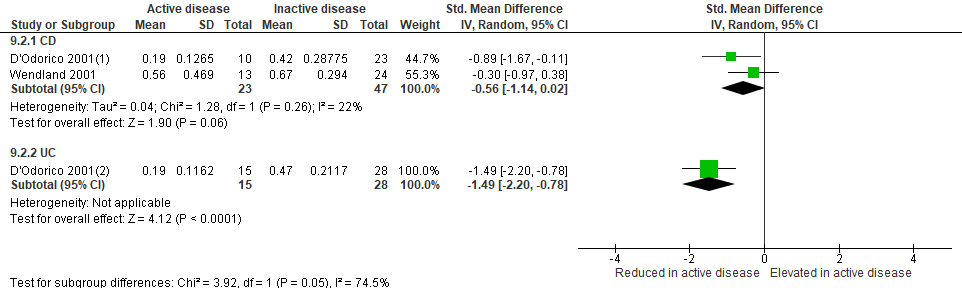
(1) cohort of patients with Crohn’s disease; (2) cohort of patients with ulcerative colitis

1. Total carotenoids


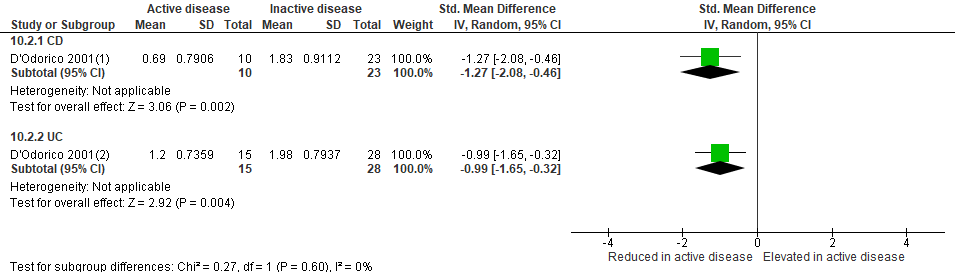
(1) cohort of patients with Crohn’s disease; (2) cohort of patients with ulcerative colitis

1. Vitamin A


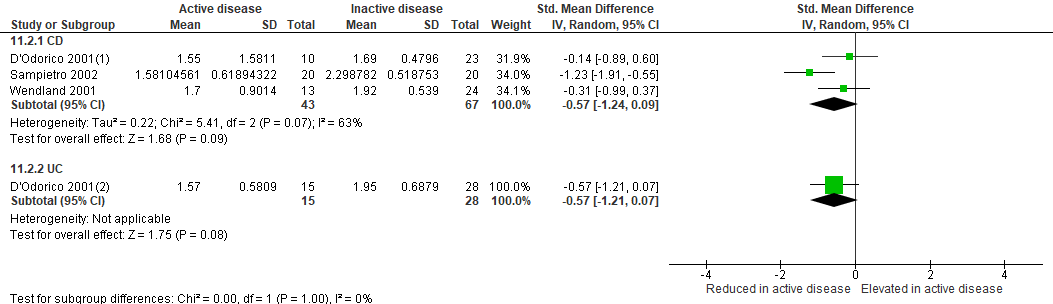
(1) cohort of patients with Crohn’s disease; (2) cohort of patients with ulcerative colitis

1. Vitamin C


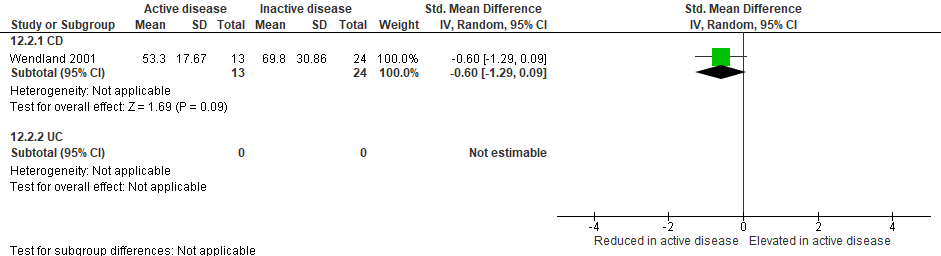


1. β-Cryptoxanthin


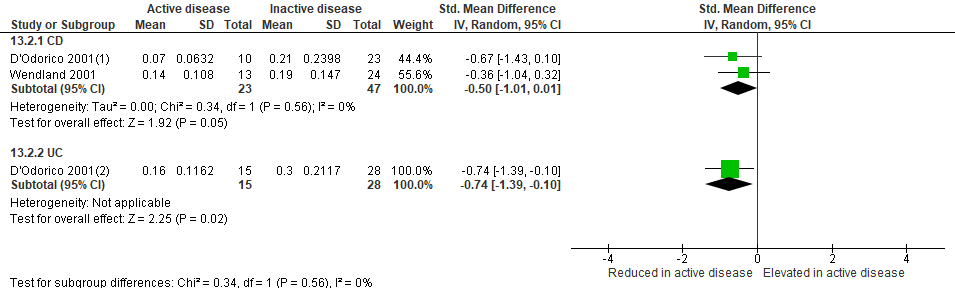
(1) cohort of patients with Crohn’s disease; (2) cohort of patients with ulcerative colitis

1. Lutein and zeaxanthin


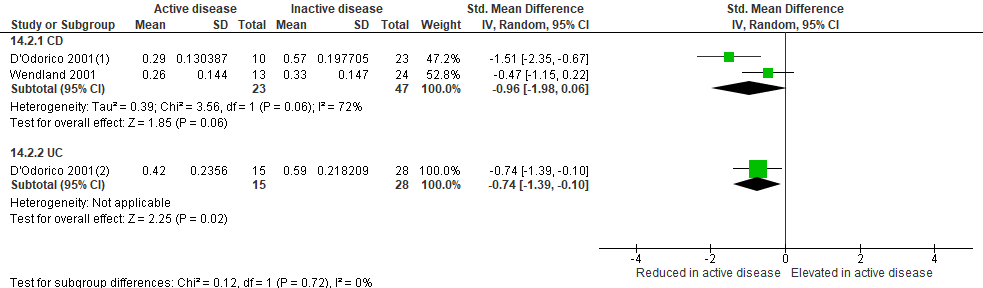
(1) cohort of patients with Crohn’s disease; (2) cohort of patients with ulcerative colitis

1. α-Carotene


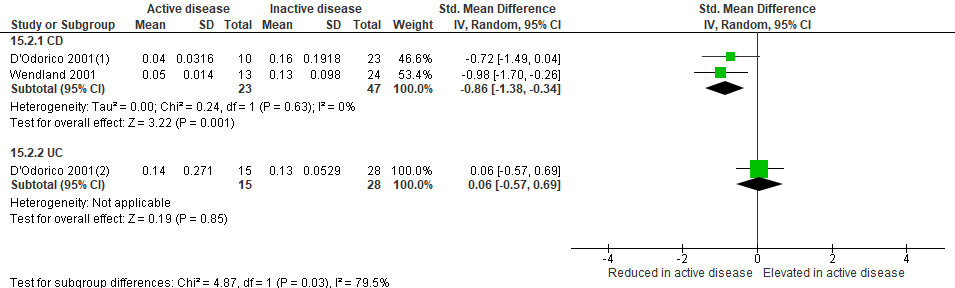
(1) cohort of patients with Crohn’s disease; (2) cohort of patients with ulcerative colitis

1. Vitamin E


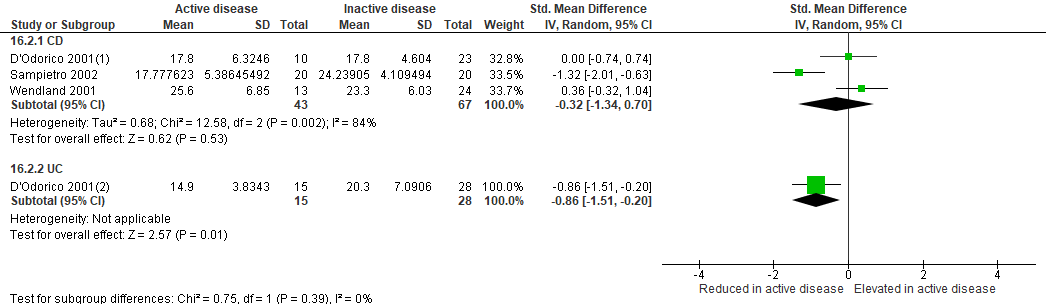
(1) cohort of patients with Crohn’s disease; (2) cohort of patients with ulcerative colitis

1. Albumin


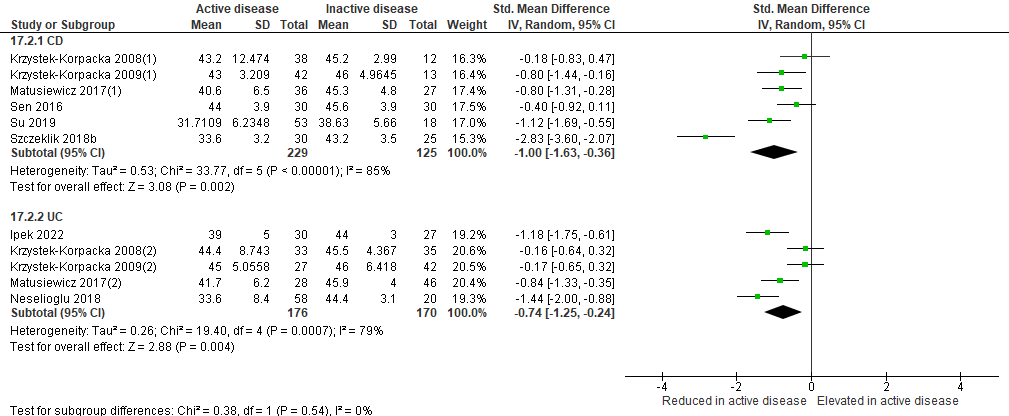
(1) cohort of patients with Crohn’s disease; (2) cohort of patients with ulcerative colitis

1. SepP


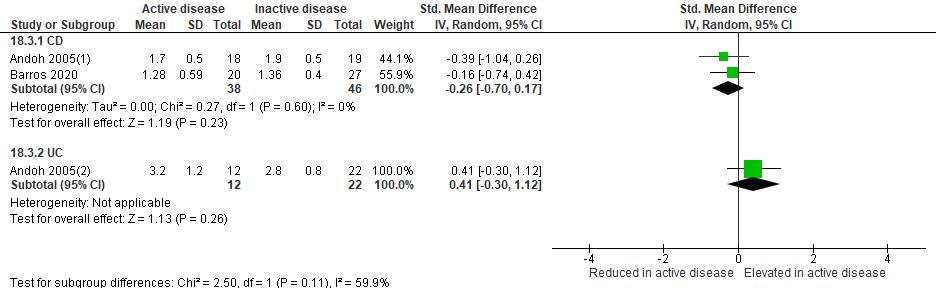
(1) cohort of patients with Crohn’s disease; (2) cohort of patients with ulcerative colitis

1. Transferrin


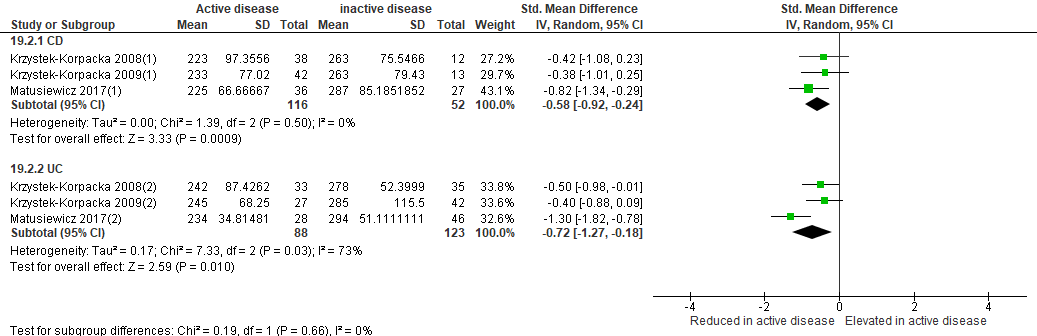
(1) cohort of patients with Crohn’s disease; (2) cohort of patients with ulcerative colitis

1. R-SH


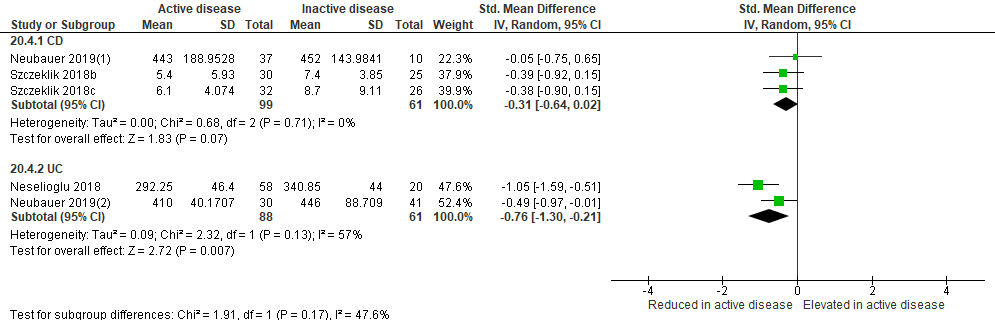
(1) cohort of patients with Crohn’s disease; (2) cohort of patients with ulcerative colitis

1. TAC


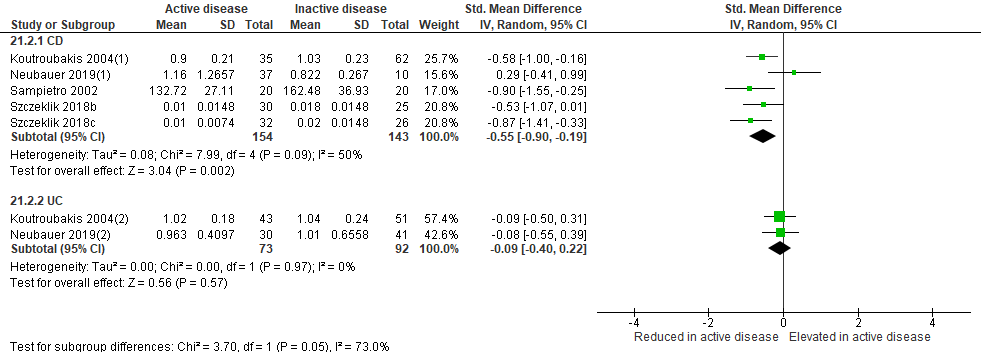
(1) cohort of patients with Crohn’s disease; (2) cohort of patients with ulcerative colitis

1. TBIL


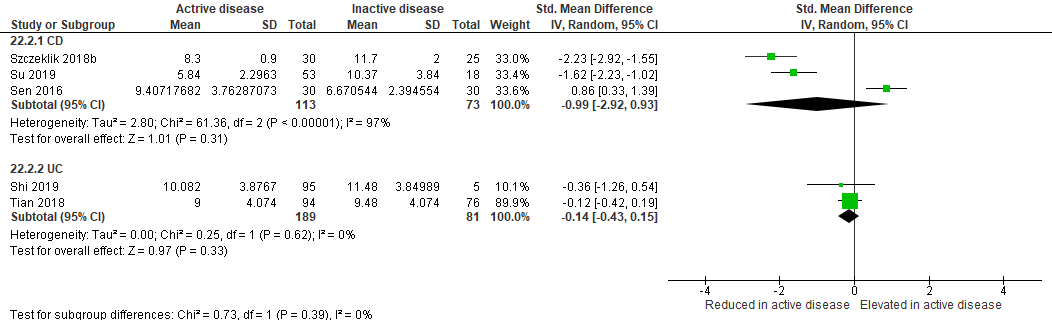


1. Se


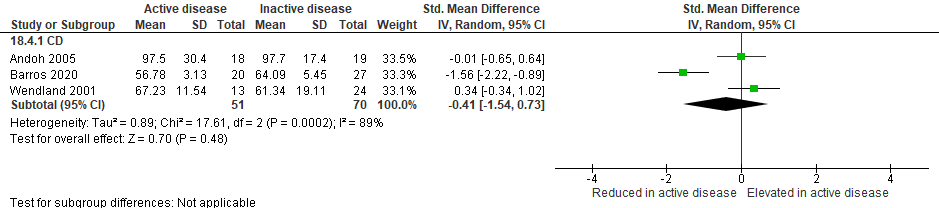


1. SUA


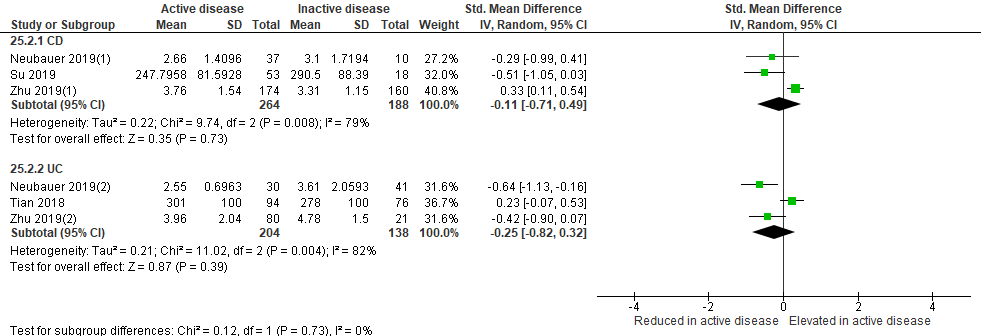
(1) cohort of patients with Crohn’s disease; (2) cohort of patients with ulcerative colitis
